# Research progress on the anti-cancer mechanisms of edible salty-flavored Chinese materia medica

**DOI:** 10.3389/fphar.2025.1598978

**Published:** 2025-06-20

**Authors:** Zhongqi Shen, Meng Yu, Zhenguo Wang

**Affiliations:** ^1^ Institute of Chinese Medical Literature and Culture, Shandong University of Traditional Chinese Medicine, Jinan, Shandong, China; ^2^ Innovative Institute of Chinese Medicine and Pharmacy, Shandong University of Traditional Chinese Medicine, Jinan, Shandong, China

**Keywords:** edible salty-flavored Chinese materia medica, traditional Chinese medicine, anti-cancer, primary prevention, health products

## Abstract

Edible salty-flavored Chinese materia medica (ESCM) refers to a category of traditional Chinese medicine (TCM) that also serve as food, characterized by their salty flavor. According to the TCM theory, ESCM can soften and disperse knots, thus potentially offering benefits for cancer prevention and treatment. With cancer remaining a major global health challenge, its primary prevention strategies, especially through dietary modification, are crucial. ESCM have recently garnered substantial attention, due to their remarkable clinical efficacy and low side-effect profile. Researches on ESCM demonstrate that they mainly function through inhibition of cancer cell proliferation, migration, and invasion, induction of cancer cell apoptosis and autophagy, regulation of the cell cycle, suppression of tumor angiogenesis, and anti-inflammatory and anti-oxidant properties. Herein, we systematically explore the well-documented ESCM’s extracts or constituents with explicit anti-cancer properties, alongside their underlying mechanisms and pathways. The review further highlights both the primary preventions and clinical trials of ESCM-related products, offering valuable insights for the development of novel dietary approaches and therapeutic interventions in cancer management.

## 1 Introduction

According to the International Agency for Research on Cancer (IARC), nearly 20 million new cancer cases and 9.7 million cancer-related deaths were projected in 2022. Current estimates indicate that approximately one in five individuals will develop cancer during their lifetime, while one in nine will succumb to the disease. Demographic projections suggest that by 2050, annual cancer incidence could rise to 35 million cases worldwide. Hence, strategic investment in prevention exhibits the potential to avert millions of future cancer diagnoses, save countless lives, and significantly alleviate the substantial socioeconomic burden (Vineis and Wild, 2014).

Primary prevention, also known as etiologic prevention, involves implementing measures to address the root causes or risk factors of a disease (or injury) before it occurs. The major goal is to reduce harmful exposure and enhance the individual’s ability to resist, thereby preventing the occurrence of the disease (or injury) or at least delaying its onset. Thus, primary prevention appears to be the fundamental strategy for the eradication of a disease (or injury). This approach is particularly effective in cancer prevention, as current knowledge indicates that one-third to one-half of cancer cases are preventable based on risk factors (McCullough and Giovannucci, 2004).

Dietary factors are indicated to underlie the substantial international variations in cancer incidence. It represents a highly effective approach to primary cancer prevention (Ko and Chiu, 2006). In addition to maintain balanced nutrition and avoid harmful dietary habits, incorporating foods with anti-cancer properties can play a pivotal role in cancer prevention and treatment. Such food can mitigate risk factors for cellular carcinogenesis or eliminate early-stage cancer cells prior to tumor formation.

Traditional Chinese medicine (TCM) utilizes various types of edible Chinese materia medica (CMM), with notable correlations between their flavors and therapeutic efficacies (Zhao et al., 2017). In TCM theory, the well-known function of salty-flavored CMM is to soften and disperse knots, making them effective in treating cancer-related tangible lumps, nodules, and masses (Shannon et al., 2021). Addressing these physical abnormalities is critical for maintaining individual’s health and preventing the pathological development from benign conditions to potentially life-threatening diseases. These highlight the significance of edible salty-flavored Chinese materia medica (ESCM) and proposes their potential roles in primary cancer prevention.

**FIGURE 1:**
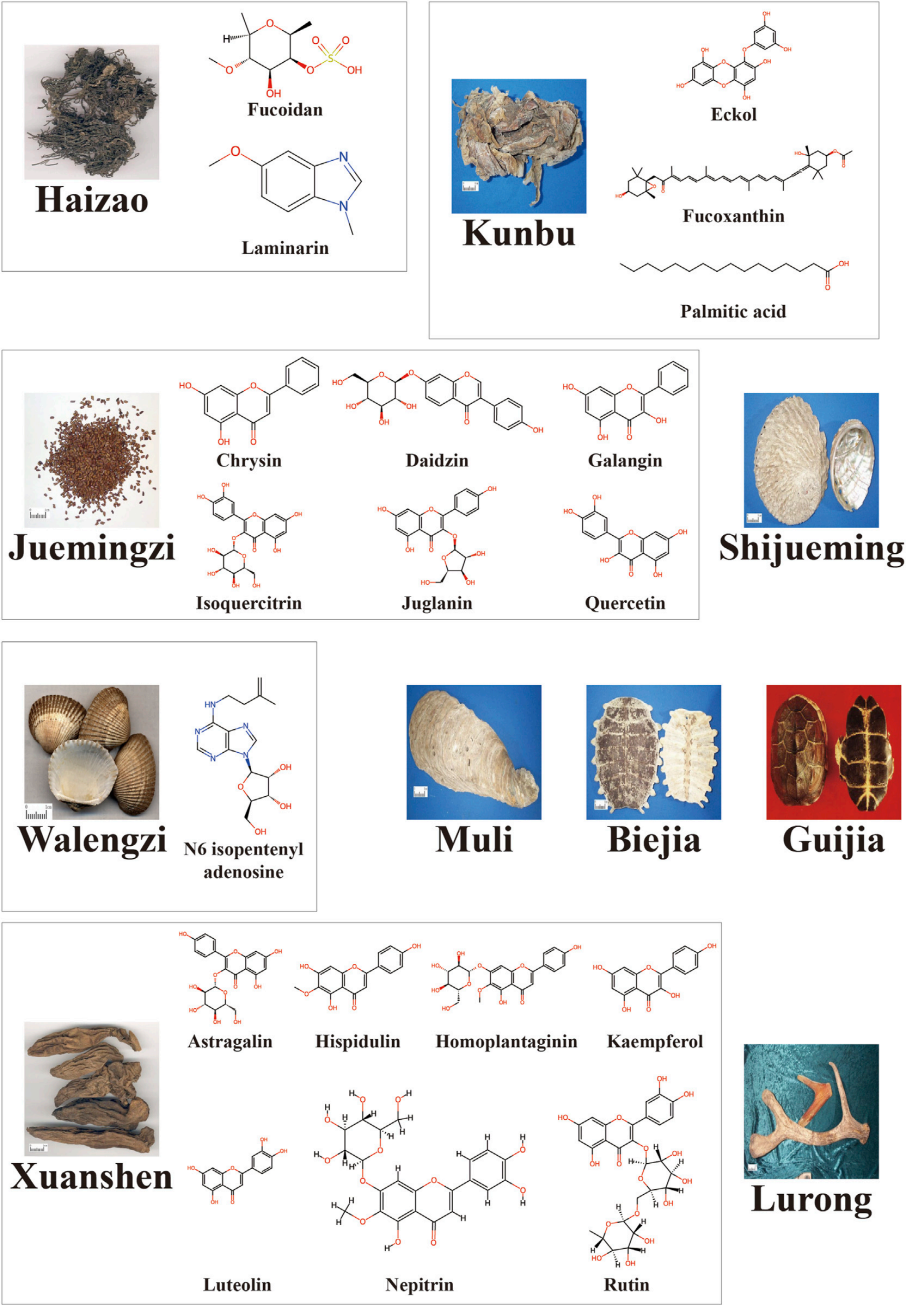
Images of ESCM and chemical structures of anti-cancer constituents. The images were downloaded from the website of Pharmaceutical Network (https://www.pharmnet.com.cn/tcm/zybb/), the chemical structures were obtained from the website of ChemSpider (https://www.chemspider.com/).

**FIGURE 2:**
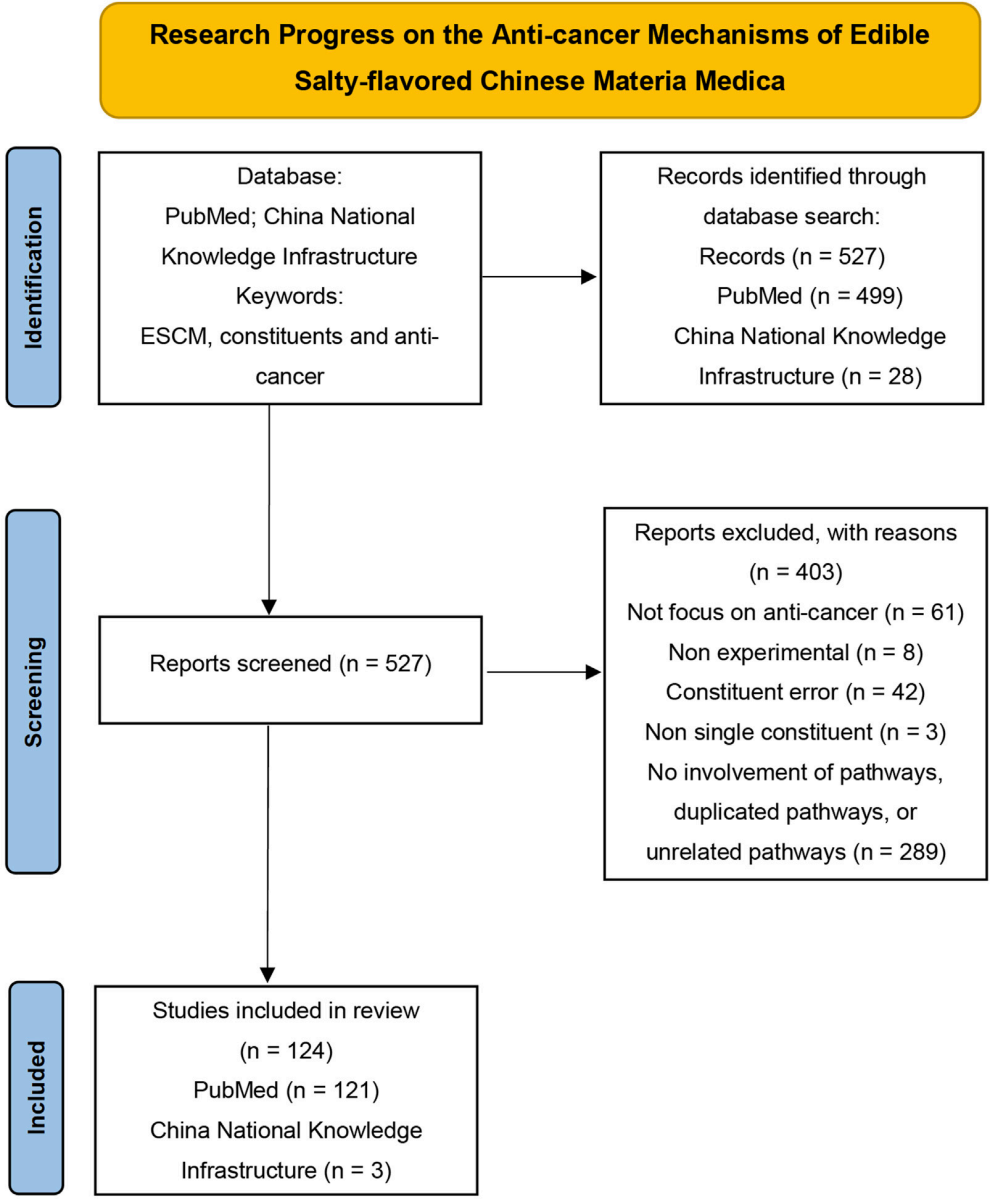
Flow diagram of the article selection process (PRISMA 2020).

Hence, we conduct a comprehensive review of well-documented ESCM and their anti-cancer properties, emphasizing primary prevention and clinical trials involving ESCM (or their constituents)-related products. The reviewed ESCM were selected from the *List of foods that double as medicine,* the *List of substances approved for usage in health supplements*, and subsequent supplementary lists, as published by the National Health Commission of the People’s Republic of China (http://www.nhc.gov.cn/), papers from China National Knowledge Infrastructure (CNKI) were also used as references. We specifically explored those extracts or constituents that have demonstrated explicit anti-cancer effects (Table 1; Figure 1) and reviewed the research progress on the mechanisms underlying their anti-cancer properties, the literature screening process is depicted in Figure 2. Notably, all the listed ESCM are commercially available as drugs or supplements, and thus clarifying their bioactive constituents and corresponding anti-cancer mechanisms can facilitate drug repurposing and dietary health applications. Overall, this review aims to provide a foundation for the future usage of ESCM in primary cancer prevention.

**TABLE 1:** Selected ESCM and their anti-cancer constituents.

Chinese materia medica	Anti-cancer constituents
Haizao *(Sargassum*)	Fucoidan; Laminarin (Liu et al., 2022; Han et al., 2020a)
Kunbu (*Laminariae Thallus*)	Eckol (Leong et al., 2022); Fucoxanthin (García-Maldonado et al., 2023); Palmitic acid (Chen et al., 2023a)
Juemingzi (*Cassiae Semen*)	Chrysin; Daidzin; Galangin; Isoquercitrin; Juglanin; Quercetin (Lewenhofer et al., 2018)
Shijueming (*Haliotidis Concha*)	Extract
Walengzi *(Arcae Concha)*	N6 isopentenyl adenosine
Muli (*Ostreae Concha*)	Extract
Biejia (*Trionycis Carapax*)	Extract
Guijia (*Testudinis Carapax Et Plastrum*)	Extract
Xuanshen (*Natrii Sulfas Exsiccatus*)	Astragalin; Hispidulin; Homoplantaginin; Kaempferol; Luteolin; Nepitrin; Rutin (Schwartz et al., 2017)
Lurong (*Cervi Cornu Pantotrichum*)	Extract

## 2 Anti-cancer

**FIGURE 3:**
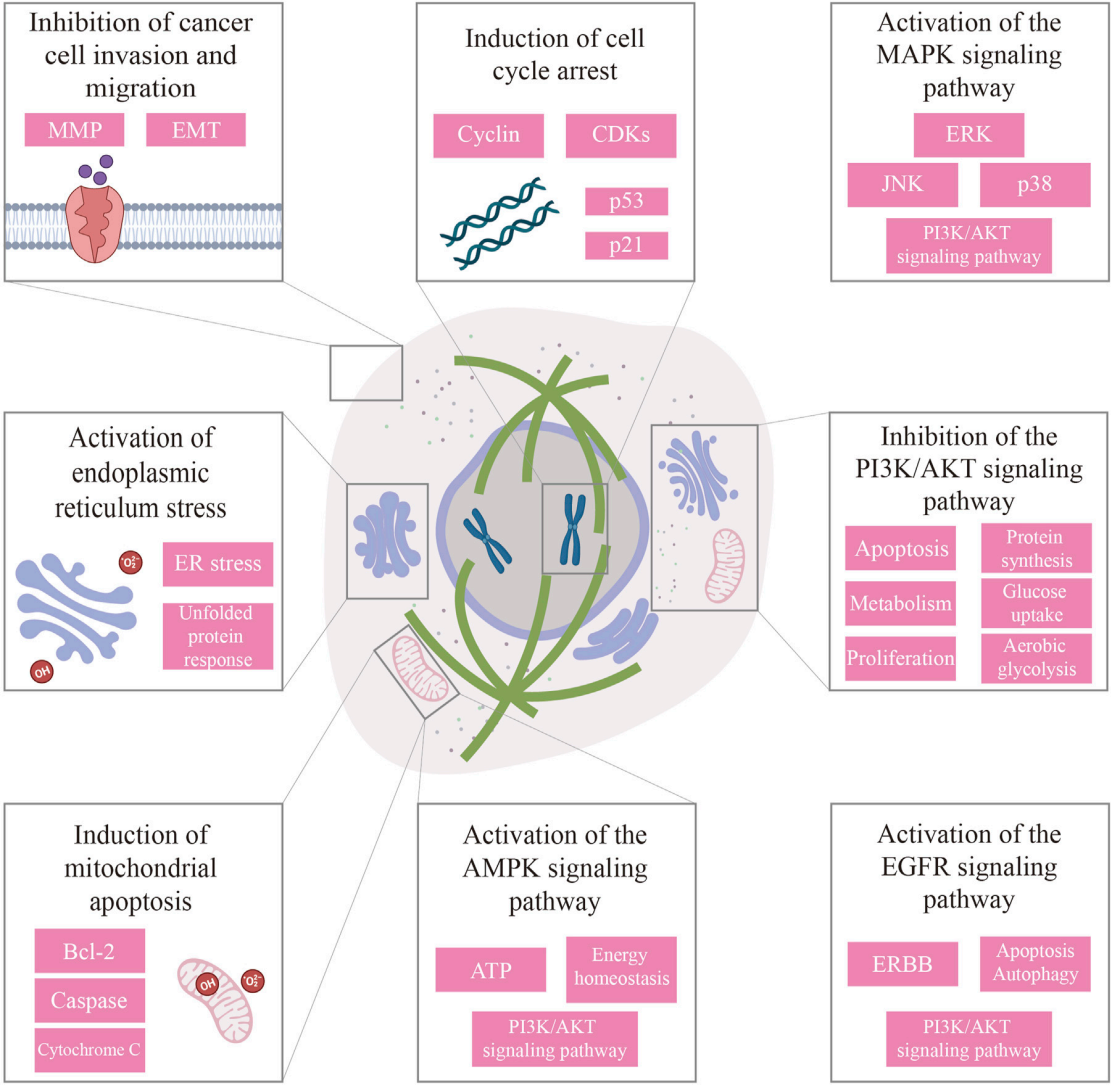
Summary of the anti-cancer mechanisms of cancer cell proliferation inhibition and apoptosis induction, and inhibition of cancer cell invasion and migration.

Excessive cell proliferation is a key prerequisite for carcinogenesis, so inhibiting cancer cell proliferation is an effective means of anti-cancer treatment. During this process, cancer cells positively compete with surrounding cells for nutrients (Icard et al., 2018). To enhance their survival and growth, cancer cells undergo metabolic reprogramming, preferentially utilizing glucose through aerobic glycolysis—a phenomenon known as the Warburg Effect (Sun et al., 2018; Jeong and Seol, 2008). In the early stage of cancer, directly inhibiting cancer cell proliferation and inducing apoptosis or autophagy through the action of various signaling pathways can be highly effective therapeutic modalities (Figure 3).

### 2.1 Induction of mitochondrial apoptosis

Mitochondria plays a crucial role in apoptotic, with its fission directly initiating the apoptosis process (Bruckheimer et al., 1998). Key regulators of apoptosis include B cell lymphoma-2 (Bcl-2) family proteins (e.g., Bcl-2, Bcl-xL, Bid, Bad, Bik, Bax) and cysteine-aspartate protease (caspase) family enzymes (Fan et al., 2005; Zhang et al., 2004). The process unfolds in a stepwise manner, beginning with the upregulation of cytochrome c (cyt c), followed by the expression of poly ADP polymerase (PARP), which serves as the cleavage substrate of caspase. This leads to the downregulation of the apoptosis inhibitor gene surviving, further facilitating the progression of apoptosis.

Chrysin can effectively inhibit the growth rate of human cervical cancer Hela cells and human breast cancer MCF-7 cells through inducing apoptosis (Geng et al., 2022; Xue et al., 2016). Additionally, human uveal melanoma cells have been reported to experience increased mitochondrial membrane permeability when exposed to chrysin. This causes the release of cyt c into the cell cytoplasm, which then activates a caspase cascade, particularly caspase-3 and caspase-9, resulting in mitochondrial impairment and triggering apoptosis (Yao et al., 2021). Similarly, daidzin can affect the permeability of mitochondrial membranes in Hela cells, thereby decreasing the expression of anti-apoptotic proteins Bcl-2 and survivin. It simultaneously increases the expression of Bax, caspase-8, and caspase-9, leading to a dose-dependent apoptosis process (Li et al., 2022; Zhang et al., 2019). The pro-apoptotic and anti-proliferative activities of eckol (a marine-derived phlorotannin) are demonstrated by the upregulation of Bax, caspase-3, and caspase-9 expression, alongside downregulation of Bcl-2 expression through *in vivo* experiments (Huang et al., 2020).

Galangin has been reported to regulate key apoptotic markers in a concentration-dependent manner in ovarian cancer cells. Specifically, it upregulates Bax protein, downregulates Bcl-2 expression, and increases cleaved caspase-3, caspase-7, caspase-8, and caspase-9 levels, that facilitating apoptosis (Liang et al., 2021). Liang et al. found that galangin can inhibit the viability of human gastric cancer MGC-803 cells while sparing normal gastric mucosal epithelial cells. This selective effect manifests as a decrease in Bcl-2 and an elevation of cleaved caspase-3 and PARP (Ha et al., 2013). Additionally, galangin can activate caspase-3 and caspase-9 in human colon cancer cells and induces apoptosis by disrupting the membrane potential of mitochondria, ultimately leading to mitochondrial dysfunction (Gao et al., 2014). Hispidulin exhibits effects similar to those of galangin. It alters mitochondrial membrane potential, decreases the Bcl-2/Bax ratio, and enhances the activation and release of cyt c and caspase-3 (Shi et al., 2021a).

Luteolin has been shown to decrease the expression of anti-apoptotic genes Bcl-2 and Bcl-xL while increasing pro-apoptotic genes such as Bax, Bad, and Bid (Kang et al., 2017). In human colon cancer HT-29 cells, luteolin treatment triggered the release of cytochrome c from mitochondria into the cytoplasm, leading to increased levels of activated caspase-9 and caspase-3 (Raina et al., 2021). Additionally, luteolin induced apoptosis by depolarizing the mitochondrial membrane potential and causing DNA damage (Budisan et al., 2019).

Kaempferol’s effects on the Bcl-2 protein family have been extensively studied. Lee et al. reported that kaempferol increased mitochondrial membrane permeability and elevated cytoplasmic cytochrome c levels in HT-29 cells. It also enhanced the levels of cleaved caspase-9, caspase-3, caspase-7, and caspase-8. Furthermore, kaempferol decreased the expression of anti-apoptotic proteins such as Bcl-xL and Bid, while upregulating pro-apoptotic proteins like Bad and Bik. Additionally, it activated cell surface death receptors, contributing to apoptosis (Lee et al., 2014; Khorsandi et al., 2017).

Cancer cells generate substantial amounts of lactate during aerobic glycolysis, which is expelled into the tumor microenvironment via the monocarboxylate transporter protein (MCT). Quercetin has been shown to significantly downregulate Bcl-2 expression and upregulate Bax expression in MCF-7 cells, thereby inducing mitochondrial apoptosis. Moreover, Amorim et al. reported that quercetin inhibits MCT expression in colorectal cancer cells, thus disrupting their glycolytic phenotype. This disruption deprives cancer cells of sufficient energy, followed by cell proliferation inhibition and apoptosis (Amorim et al., 2015; Thorpe et al., 2015).

### 2.2 Intervention in the PI3K/AKT and its associated signaling pathways

The phosphatidylinositol 3-kinase/protein kinase B (PI3K/Akt) signaling pathway is a well-established biological process involving the regulation of diverse signalings such as apoptosis, metabolism, cell proliferation and growth, protein synthesis, transcription, glucose uptake, and aerobic glycolysis in cancer cells (Fresno Vara et al., 2004; Fruman et al., 2017). Activation of PI3K within this pathway is driven by oncogenes and growth factor receptors, and its heightened activity is frequently recognized as a hallmark of cancer (Chen et al., 2017). Intervention in the adenosine monophosphate-activated protein kinase (AMPK), mitogen-activated protein kinases (MAPK), and epidermal growth factor receptor (EGFR) signaling pathways, as well as inhibition of the PI3K/Akt pathway through crosstalk effects, are essential for achieving anti-cancer effects.

#### 2.2.1 Inhibition of the PI3K/AKT signaling pathway

Most listed active constituents address the functions to suppress the PI3K/AKT signaling pathway. Juglanin has been reported to suppress the PI3K/AKT signaling pathway, resulting in a reduction of anti-apoptotic proteins (Bcl-2 and Bcl-xL) and an increase in pro-apoptotic proteins (Bax and Bad). These enhance the cleavage of caspase-3 and PARP, thereby promoting apoptosis in cancer cells. Furthermore, the formation of autophagic vacuoles and upregulation of autophagy-related genes in juglanin-treated cells indicate that juglanin also induces cellular autophagy (Yang et al., 2023a). Astragalin downregulates the phosphorylation level of PI3K/Akt signaling pathway-related proteins and activates autophagy in mouse hippocampal neuron HT22 cells (Wang and Tang, 2017). *In vivo* and *in vitro* experiments have shown that galangin can promote caspase-3 expression by inhibiting the PI3K/AKT signaling pathway (Hsu et al., 2022).

#### 2.2.2 Activation of the AMPK signaling pathway

AMPK is a crucial energy sensor that monitors changes in AMP/ATP or ADP/ATP ratios and regulates metabolic processes, playing a vital role in maintaining cellular energy homeostasis (Nitulescu et al., 2018). Additionally, it can modulate glucose and lipid metabolism by responding to fluctuations in nutrient and extracellular energy levels (Keerthana et al., 2023). Activation of AMPK and its downstream signaling cascades orchestrates the dynamics of bioenergetics and metabolism in tumor cells. Substantial evidence supports the inhibitory role of AMPK activation in tumorigenesis and progression, suggesting that targeting the AMPK signaling pathway could provide a promising strategy for cancer therapy (Zeb et al., 2024). Astragalin activates the AMPK signaling pathway, and inhibits the aerobic glycolysis and proliferation of human breast cancer MDA-MB-231 cells through AMPK-mediated metabolic regulation (Wang et al., 2015). Hispidulin could potentiate the anti-tumor activity of temozolomide (TMZ) in glioblastoma by activating the AMPK signaling pathway (Franco-Juárez et al., 2022). Transcription factor EB (TFEB), a master regulator of autophagy and lysosomal biogenesis, is upregulated by homoplantaginin through AMPK/TFEB pathway activation (Fan et al., 2023; Wu et al., 2017). Isoquercitrin reduces viability and promotes apoptosis in T24 bladder cancer cells via AMPK-mediated metabolic dysfunction and caspase-dependent apoptosis (Zeng and Chen, 2022). The sirtuin (SIRT) protein family critically regulates mitochondrial biosynthesis and intervenes in mitochondrial function via an AMPK-dependent mechanism (Guo H. et al., 2021). Guo et al. reported that quercetin elevates the SIRT1 expression level and the AMPK phosphorylation level in a dose-dependent manner, thereby activating the SIRT1/AMPK axis to trigger mitochondria-dependent apoptotic pathway in human A549 cells and H1299 cells, to treat the non-small cell lung cancer (NSCLC) (Lee et al., 2020).

#### 2.2.3 Activation of the MAPK signaling pathway

MAPK are pivotal signaling mediators that respond to diverse stimuli, including physiological signals (e.g., hormones, cytokines, and growth factors) and stress-related cues (e.g., endogenous stress and environmental perturbations). The MAPK family comprises three major subfamilies: ERK (a pro-survival kinase activated by mitogenic signals), and the stress-responsive MAPKs, JNK and p38 (Sugiura et al., 2021). Sustained ERK activation can significantly trigger tumor cell death under specific conditions (Dhanasekaran and Reddy, 2008). JNK, often termed the stress-activated protein kinase, primarily regulates stress-induced apoptosis and damage response. Mechanistically, JNK activation promotes apoptotic signaling by either upregulating pro-apoptotic genes via trans-activation of specific transcription factors or by directly modulating mitochondrial apoptotic pathways through phosphorylation-dependent regulation of Bcl-2 family proteins (Sui et al., 2014). Notably, the p38 and JNK pathways exhibit functional interaction in the induction of apoptosis and autophagy processes, with their interplay dictating context-dependent cell fate decisions (Pichichero et al., 2011). Chrysin induces caspase-dependent apoptosis in both human and murine melanoma cells through activation of ERK and p38 MAPK signaling pathways (Kim DA. et al., 2012). Galangin-triggered cell apoptosis was characterized by DNA breaks, caspase-3/9 activation, PARP cleavage, and coordinated activation of the expression of MAPK kinases (ERK and JNK) in human gastric cancer SNU-484 (Sabbah et al., 2020).

#### 2.2.4 Activation of the EGFR signaling pathway

EGFR is a member of the ERBB family of receptor tyrosine kinases, regulates critical cellular processes, including proliferation, differentiation, division, survival, and oncogenesis through its singling cascade (Jackson and Ceresa, 2017; Nam et al., 2016). Additionally, EGFR overexpression can induce receptor-mediated apoptosis or autophagy, commonly through intersecting with core apoptotic and autophagic pathways (Wu and Zhang, 2020; Anson et al., 2018). Anson et al. demonstrated that luteolin, in the presence of epidermal growth factor (EGF), can induce caspase and PARP cleavages, to suppresses the proliferation of glioblastoma cells (Shao et al., 2012).

#### 2.2.5 Crosstalk of signaling pathways associated with PI3K/Akt

Multiple constituents of ESCM can act on the upstream or downstream pathways of PI3K/Akt, ultimately intervening in the PI3K/Akt pathway through crosstalk effects. For example, chrysin and kaempferol can suppress the Akt/mTOR pathway by significantly activating the AMPK signaling pathway (Filomeni et al., 2010; Boo et al., 2013). Fucoidan, luteolin, and quercetin can also inhibit the expression of PI3K/Akt by activating the MAPK pathway (Lu et al., 2017; Granato et al., 2017; Han et al., 2017). Luteolin also reduces phosphorylated Akt and mTOR levels through activation of the EGFR, to inhibit its downstream Akt/mTOR signaling pathway (Shao et al., 2012). Among them, fucoidan (Hyun et al., 2009; Shi et al., 2021b), luteolin (Lin et al., 2015; Glaviano et al., 2023), kaempferol (Wang R. et al., 2023; Carnero et al., 2008; Xie et al., 2013; Jia et al., 2018), and quercetin (Fu et al., 2021) have all been proven to possess the ability to directly intervene in the PI3K/Akt pathway. To clarify the ESCM with both direct and indirect effects on the PI3K/Akt pathway, we constructed Table 2 for presentation and comparison.

**TABLE 2:** ESCM with both direct and indirect effects on the PI3K/Akt pathway and their indications.

ESCM	Direct effects	Indirect effects		
		Through AMPK pathway	Through MAPK pathway	Through EGFR pathway
Fucoidan	Bladder cancer Colon cancer	—	Prostate cancer	—
Luteolin	Breast cancer; Choroidal melanoma	—	Gastric cancer	Glioblastoma
Kaempferol	Non-small cell lung cancer; Bladder cancer	Cervical cancer	—	—
Quercetin	Breast cancer	—	Primary effusion lymphoma	—

### 2.3 Activation of endoplasmic reticulum stress

Endoplasmic reticulum (ER) is a critical organelle for protein synthesis and maturation. Various physiological and pathological conditions can lead to an accumulation of misfolded proteins in the ER lumen—a conserved adaptive reaction known as ER stress, and triggering the unfolded protein response (UPR) (Fu et al., 2021; Bhat et al., 2017). Persistent ER stress may induce apoptosis or autophagy as compensatory mechanisms to restore proteostasis (Chen et al., 2014). Targeting ER stress cascades has emerged as a promising anti-cancer strategy. For instance, Chen et al. demonstrated that fucoidan can exert anti-tumor effects by modulating ER stress-related apoptosis (Liu et al., 2017)^.^ Similarly, quercetin participates in the mitochondrial apoptotic pathway through Bcl-2-regulated ER stress while concurrently activating cytoprotective autophagy in human ovarian cancer cells (Liu et al., 2023). Isoquercitrin also promotes immunogenic cell death (ICD) in human gastric cancer cells through ER stress activation (Hu et al., 2019).

Sustained ER stress upregulates the C/EBP homologous protein (CHOP), a predominant pro-apoptotic transcription factor in ER, triggering downstream cascade responses (Kim et al., 2018). Kaempferol, for example, activates the JNK/CHOP signaling axis to induce ER stress and autophagy (Ibrahim et al., 2019). Additionally, glucose-regulated protein (GRP) 78 and GRP94 are crucial ER chaperone proteins, that facilitating the refolding or degradation of misfolded proteins via ER-associated degradation (ERAD) signaling pathways (Eletto et al., 2010; Su et al., 2013). Galangin enables the prolonged ER stress in hepatocellular carcinoma (HCC) by elevating GRP78, GRP94, and CHOP levels, thereby suppressing proliferation (Song W. et al., 2017). Moreover, galangin enhances TRAIL (tumor necrosis factor-related apoptosis-inducing ligand) sensitivity by upregulating CHOP-dependent DR4 (death receptor 4) activity and activating AMPK signaling. This dual mechanism promotes caspase-3-mediated apoptosis in breast cancer cells (Kuang et al., 2023).

Ferroptosis, an iron-dependent form of programmed cell death driven by lipid peroxidation, holds therapeutic potential. Palmitic acid (PA) suppresses colorectal cancer cell viability *in vitro* and *in vivo* by inducing ER stress. Mechanistically, PA disrupts intracellular iron homeostasis via ER calcium release, upregulates transferrin (TF)-mediated iron transport, and ultimately triggers ferroptotic death through iron overload (Engeland, 2022).

### 2.4 Induction of cell cycle arrest

Cell cycle arrest, achieved by downregulating cyclins and cyclin-dependent kinases (CDKs), represents a key anti-cancer strategy. Tumor suppressors such as p53 (which induces apoptosis and cycle arrest) and p21 (a CDK inhibitor and primary transcriptional target of p53) are central to this process (Weng et al., 2005). Integrating cell cycle arrest with apoptosis or autophagy often enhances therapeutic efficacy.

Chrysin induces G1-phase arrest in rat glioma C6 cells by activating p38 MAPK, which drives p21 accumulation and suppresses CDK2/CDK4 activity in a dose- and time-dependent manner (Yang et al., 2014a). Hispidulin has been indicated with diverse mechanistic insights in cell cycle arrest: in gastric cancer AGS cells, it sustains NAG-1 (NSAID-activated gene-1) expression through ERK activation, followed by the downregulation of cyclin D1/E, and eventually induces G1/S arrest with apoptosis (Yu et al., 2013; Lin et al., 2010); in glioblastoma multiforme (GBM) cells, it activates AMPK, thereby inhibiting the downstream mTOR expression, and thus upregulating p53/p21 to block G1 progression (Ishikawa et al., 2008). Fucoxanthin downregulates cyclin D1/2 and CDK4/6 to trigger G0/G1 arrest while simultaneously promoting apoptosis via caspase-3/8/9 activation, PARP cleavage, and suppression of anti-apoptotic proteins Bcl-2, Bcl-xL, and survivin, which has been validated *in vitro* and *in vivo* (Kim KN. et al., 2013; Liu et al., 2018).

Galangin suppresses PI3K/Akt signaling in both MCF-7 cells and human nasopharyngeal carcinoma cells, downregulating cyclin D3/B1 and CDK1/2/4 while elevating p21/p53 levels. This results in cell cycle arrest in the S phase along with mitochondrial apoptosis (Lee et al., 2018; Wu et al., 2018). Kaempferol exhibits selective toxicity toward EJ bladder cancer cells compared to normal bladder cells. It inhibits p-AKT, cyclin D1, CDK4, Bid, and Bcl-xL while upregulating p53/p21/Bax to induce S-phase arrest and apoptosis (Huang et al., 2013). In hepatocellular carcinoma SK-HEP-1 cells, kaempferol triggers G2/M arrest via cyclin B/CDK1 downregulation and activates AMPK-mediated autophagy by suppressing Akt/mTOR (Wang QZ. et al., 2023). Furthermore, the differential metabolite of walengzi, N6-isopentenyladenosine, induces S/G2 arrest and apoptosis in thyroid cancer TPC-1 cells (Sun et al., 2017). Exposure to juglanin activates JNK in human breast cancer cells, leading to the activation of cleaved caspase-3/8/9, and induces autophagy (evidenced by autophagosome formation). This combined effect results in G2/M phase block, apoptosis, and autophagy, effectively inhibiting cancer cell proliferation (Deng et al., 2013).

Quercetin exhibits broad-spectrum cell cycle interference capability across cancer types. In MCF-7 cells, quercetin induces G0/G1 arrest by downregulating survivin mRNA to promote apoptosis (Mu et al., 2007); in HepG2 hepatocellular carcinoma, it elevates p53 and p21 to block G1 progression (Kim H. et al., 2013). Additionally, quercetin activates caspase-3/7/9, promotes PARP degradation, stimulates JNK, and increases the expression of p53. The subsequent translocation of p53 to mitochondria triggers the release of cyt c into the cytoplasm, thereby inducing mitochondrial apoptosis and modulating cell death in human glioma U373MG cells (Moon et al., 2003). Quercetin can further suppress ERK activity, followed by downregulation of cyclins and CDKs, and upregulates the CDK inhibitor p21 to enforce G1-phase arrest (Jeong et al., 2009). Its cell cycle interference spans multiple phases: in ovarian cancer SKOV-3 cells, it downregulates cyclin B1 and CDK1—key drivers of G2/M progression—thereby blocking the G0/G1-to-G2/M transition and inducing apoptosis (Ren et al., 2015; Ong et al., 2004). In nasopharyngeal carcinoma HK1 and CNE2 cells, quercetin exhibits synergic effects regarding cell cycle arrest and apoptosis, wherein it regulates pro-apoptotic mediators (Bad, caspase-3, and caspase-7) to induce cytotoxic effects while concurrently arresting cells in G0/G1 or G2/M phases (Pastushenko and Blanpain, 2019).

## 3 Anti-metastatic/immunomodulatory

Metastasis is the most lethal manifestation of cancer, accounting for the majority of cancer-related deaths. Unlike localized tumors, metastatic ones are systemic and frequently exhibit therapy resistance, underscoring the urgency and the potential of targeting metastatic pathways. During this process, tumor cells acquire invasive traits to dissociate from the primary tumor, migrate through surrounding tissues, and colonize distant organs—a progression driven by dynamic interactions with immune, stromal, and extracellular matrix components (Zhao et al., 2023). Emerging evidence highlights bioactive constituents from ESCM as promising anti-metastatic agents (Figure 3). These constituents interfere with metastasis-associated signaling networks, including pathways regulating matrix metalloproteinase (MMP) activity, epithelial-mesenchymal transition (EMT), and micro-environmental crosstalk (Zheng et al., 2024; Deryugina and Quigley, 2006). Other effective intervention methods include targeting VEGF-mediated tumor angiogenesis, anti-inflammatory and anti-oxidant.

### 3.1 Inhibition of MMP expression

Matrix metalloproteinases (MMPs), a family of proteolytic enzymes critically implicated in tumor metastasis, drive cancer progression by degrading extracellular matrix (ECM) components and thus facilitating invasive cell behavior, with elevated MMP expression strongly correlating with aggressive metastatic phenotypes (Yang et al., 2014b). Targeting MMP activity through pharmacological or genetic interventions has emerged as a validated strategy to suppress cancer cell invasion, as exemplified by bioactive compounds derived from natural sources. Chrysin selectively downregulates MMP-10, demonstrating potent anti-metastatic effects in triple-negative breast cancer (TNBC) models (Han M. et al., 2016), whereas quercetin dose-dependently reduces MMP-2/9 levels to block colorectal Caco-2 cell motility (Fu et al., 2021; Chung et al., 2013). Similarly, fucoxanthin decreases MMP-9 expression and secretion, and further broadens its mechanistic impact through downregulating cell-surface glycoproteins and chemokine receptors essential for adhesion and invasion (Delma et al., 2015). Fucoidan, in addition to suppressing MMP-2/9 activity in pancreatic cancer cells, synergistically inhibits proliferation and induces apoptosis (Han et al., 2015). This effect might be attributed to its dose-dependent inhibition of the PI3K/Akt/mTOR pathway that reducing MMP-2 expression, as observed in HT-29 cells (Monsef-Esfahani et al., 2014) Nepitrin, even at low concentrations, directly inhibits the enzymatic activity of MMP-2 and MMP-9, thereby attenuating proteolytic ECM remodeling (Yao et al., 2019), while luteolin suppresses MMP-2/9 expression to impair migration and invasion of cells of colorectal cancer and breast cancer (Feng et al., 2020; Li et al., 2015). Kaempferol exemplifies multi-targeted efficacy: by downregulating ERK, JNK, and p38 expression, it suppresses MAPK signaling to reduce MMP-2/9 activation, thereby inhibiting adhesion, migration, and invasion in breast cancer MDA-MB-231 cells. Critically, *in vivo* studies confirm kaempferol’s translational potential, as it disrupts the MAPK/MMP-9 signaling cascade to prevent lung metastasis in murine melanoma B16F10 models (Chen et al., 2013; Lamouille et al., 2014). Collectively, these findings underscore MMP suppression as a cornerstone of anti-metastatic therapy, with natural compounds offering multifaceted mechanisms to block ECM degradation, impair cell motility, and synergize with apoptotic and proliferation pathways.

### 3.2 Suppression of EMT process

EMT is a reversible cellular reprogramming process enabling epithelial cells to acquire migratory mesenchymal traits. It is a critical driver of cancer metastasis that intimately correlates with advanced tumor stage, therapeutic resistance, and poor clinical prognosis (Zhao et al., 2023). During EMT, diminished cell adhesion—marked by downregulated E-cadherin and upregulated N-cadherin and vimentin—enhances cancer cell invasiveness and migration ability, facilitating detachment from primary tumors and dissemination to distant organs (Zhang and Weinberg, 2018). Furthermore, EMT synergizes with MMP overexpression to degrade ECM barriers, amplifying metastatic potential (He et al., 2019).

Natural compounds targeting the EMT process demonstrate considerable therapeutic promise. Chrysin reverses EMT in TNBC by restoring E-cadherin and suppressing vimentin, directly impeding metastasis (Han M. et al., 2016). Fucoidan-treated rat serum modulates MCF-7 cells by upregulating E-cadherin and downregulating MMP-9, as it can significantly inhibit migration and invasion via attenuating EMT, while concurrently enhancing apoptosis (Lin et al., 2017)^.^ Similarly, luteolin upregulates E-cadherin and downregulates mesenchymal markers such as N-cadherin, and vimentin expression, thereby restoring the epithelial phenotype of TNBC cells (Jo et al., 2015). In NSCLC A549 cells, kaempferol effectively blocks TGF-β1-induced EMT and cell metastasis by reestablishing E-cadherin expression and inhibiting MMP-2 and TGF-β1 upregulation (Zhu et al., 2021).

The PI3K/Akt/mTOR pathway is a central upstream regulator of EMT, that drives metastasis by promoting cadherin switching, vimentin overexpression, and MMP-2/9 activation. Pharmacological inhibition of PI3K, Akt, and mTOR, at both protein and mRNA levels, reversing EMT and hindering cancer cell proliferation, invasion, and migration. For example, biejia extract could inhibit PI3K/Akt/mTOR signaling in MDA-MB-231 breast cancer cells, attenuating EMT and metastatic behaviors (Chen et al., 2018). Chen et al. found that luteolin demonstrates multi-target efficacy by disrupting RPS19-activated EMT in cutaneous squamous cell carcinoma via Akt/mTOR pathway blockade (Yang et al., 2022). Additionally, daidzin suppresses PI3K/Akt/mTOR pathway and TGF-β expression, effectively impeding EMT and reducing invasiveness in colon (SNU-C2A) and prostate (DU145, PC-3) cancers (Wei et al., 2021). These findings manifest PI3K/Akt/mTOR inhibition as a potent strategy to counteract EMT-driven metastasis.

### 3.3 Targeting VEGF-mediated tumor angiogenesis

**FIGURE 4:**
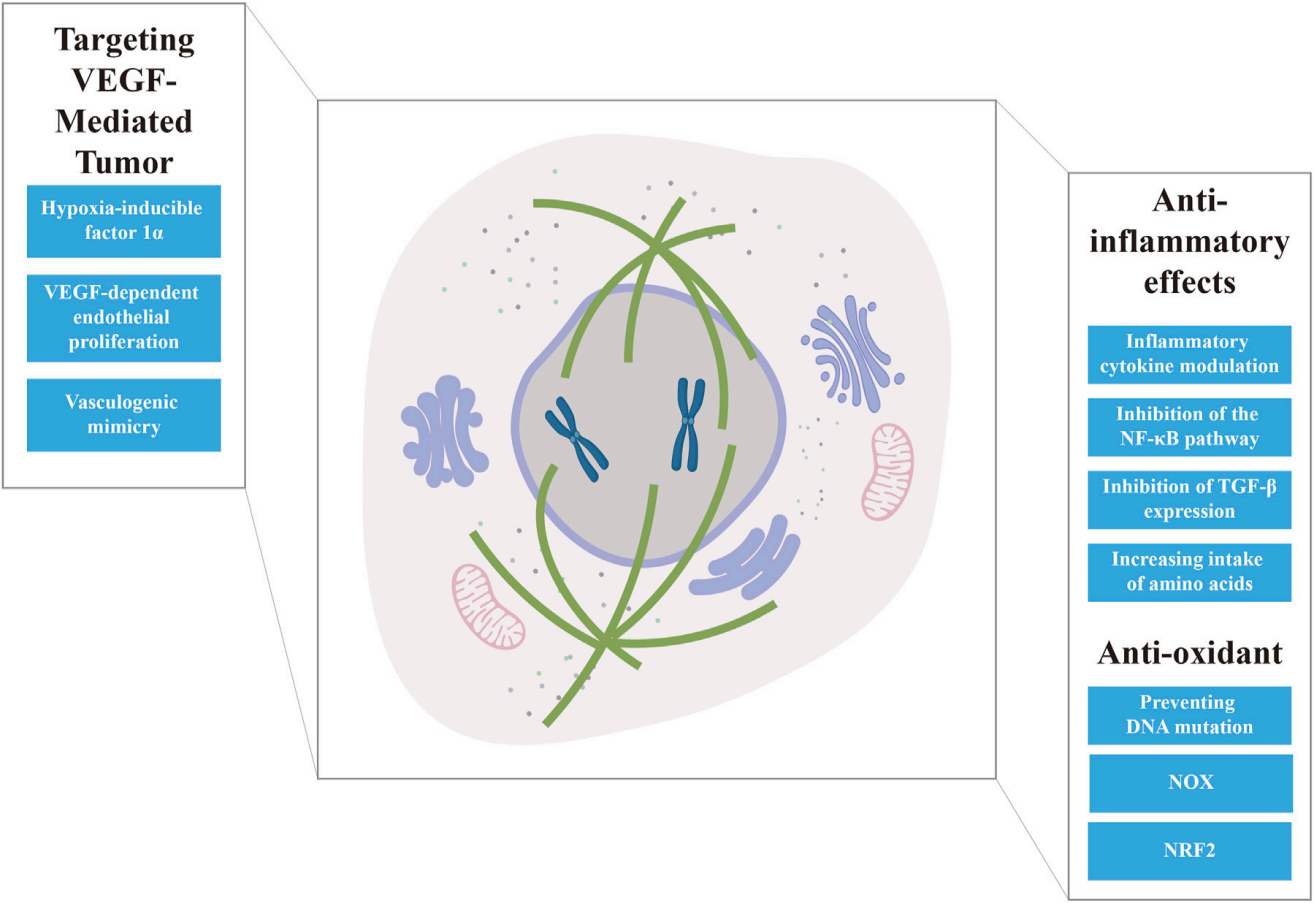
Summary of the anti-cancer mechanisms of targeting VEGF-mediated tumor angiogenesis, and anti-inflammatory and anti-oxidant effects.

Vascular endothelial growth factor (VEGF) drives tumor angiogenesis through hypoxia-inducible factor 1α (HIF-1α)-mediated transcriptional regulation, a process amplified by oncogene signals, growth factors, and hypoxic stress. Tumors exceeding 1–2 mm in diameter require neovascularization to overcome diffusion-limited nutrient supply. This further prompts VEGF-dependent endothelial proliferation and vasculogenic mimicry (VM), where tumor cells self-organize into functional microvascular networks, bypassing traditional angiogenic pathways (Rashid et al., 2021). HIF-1α serves as the molecular linchpin, simultaneously upregulating VEGF while activating PI3K/Akt/mTOR and MAPK signaling cascades, thereby creating a feedforward loop that sustains tumor vascularization and progression (DeNicola and Cantley, 2015; Carmeliet, 2005). This mechanistic overlap posits VEGF suppression as a strategic therapeutic frontier, particularly given VM’s resistance to conventional anti-angiogenic therapies (Figure 4) (Mabeta and Steenkamp, 2022; Liu et al., 2016).

The previously documented constituents demonstrate multi-target efficacy against VEGF-driven tumor angiogenesis: fucoidan exhibits pan-inhibitory efficacy across diverse malignancies, including multiple myeloma (RPMI-8226, U266) and breast cancer (4T1), which suppresses VEGF expression to potently ameliorate tumor neoangiogenesis (Xue et al., 2012; Liu et al., 2012). Mechanically, it disrupts both microvascular proliferation and vascular network patterning, while concurrently suppressing lymphangiogenic signaling pathways, thereby reducing lymphatic metastasis incidence in preclinical models (Yang et al., 2016; Huang et al., 2015). Galangin inhibits angiogenesis in ovarian carcinoma OVCAR-3 cells via VEGF suppression and prevents neovascularization through blockade of the Akt/HIF-1α pathway (He et al., 2011). Similarly, hispidulin interferes with the VEGF receptor-2 (VEGFR-2) mediated PI3K/Akt/mTOR signaling axis to inhibit the growth of pancreatic tumors and angiogenesis (Hasan and Fischer, 2023). Parallel targeting of Notch signaling, a HIF-1α/Akt-regulated endothelial specification pathway, enhances anti-angiogenic efficacy, as evidenced by luteolin-enabled reduction in gastric cancer VM formation via Notch1/VEGF crosstalk inhibition (Zang et al., 2017; Lirdprapamongkol et al., 2013). Chrysin eliminates hypoxia-induced VEGF transcription in mouse breast cancer model with decreased pulmonary metastasis (Coussens and Werb, 2002). These indicate phytochemical strategies as viable solutions to overcome tumor vascular plasticity.

### 3.4 Anti-inflammatory

Chronic inflammation constitutes a pivotal driver of carcinogenesis, with approximately 20% of malignancies arising from infection-associated or inflammation-prone microenvironments (Quail and Joyce, 2013). Sustained inflammatory insults induce cumulative DNA damage and epigenetic reprogramming, fostering malignant transformation through two synergistic mechanisms: (1) persistent immune cell activation (neutrophils, macrophages, lymphocytes) and (2) tumor co-option of inflammatory mediators (IL-6, TNF-α, COX-2) as metastatic accelerants. The tumor microenvironment (TME), comprising immune cells (e.g., myeloid-derived suppressor cells, tumor-associated macrophages), stromal fibroblasts, and vascular endothelial cells, exploits this inflammatory circuitry by repurposing cytokines like IL-1β and IFN-γ to activate invasion-associated MMPs and immune-evasion pathways (Figure 4) (Eggert and Greten, 2017; Wang et al., 2024). Consequently, therapeutic strategies targeting inflammatory pathways demonstrate dual efficacy of attenuating chronic tissue damage that predisposes to malignant transformation and disrupting pro-tumorigenic cytokine networks within TME (Chen et al., 2016).

#### 3.4.1 Inflammatory cytokine modulation

Immunomodulatory constituents enhance innate immune surveillance. Biejia and shijueming extracts can both augment macrophage phagocytic activity to reduce neutrophil infiltration and immune response (Chen et al., 2021). Additionally, biejia extract simultaneously elevates pro-inflammatory cytokines (IL-2, IL-4, IFN-γ, and TNF-α) and immunosuppressive IL-10, suggesting a rebalancing of immune homeostasis. Furthermore, administration of biejia extract and muli hydrolysate manifests enhanced immune ability correlating with increased thymic indices and splenic lymphocyte proliferation (Wang et al., 2010; Kim TH. et al., 2012). Apart from these, eckol activates macrophage populations while inhibiting leukocyte adhesion through integrin modulation (Huang et al., 2020; Suh et al., 2007). Lurong hydrolysate can also significantly reduce the expression of IFN-γ and TNF-α in mice models (Granato et al., 2017).

Targeted cytokine suppression with constituents can disrupt tumor-immune crosstalk. Quercetin uniquely facilitates immunogenic cell death by upregulating surface calreticulin, improving immune recognition of apoptotic cells while reducing IL-6 and IL-10 secretion (Chen et al., 2019). Juglanin demonstrates dual efficacy, reducing chronic ultraviolet radiation b (UVB)-induced pro-inflammatory cytokine release and IL-1β-driven MMP production, thereby inhibiting ECM degradation and remodeling TME (Rehman et al., 2013). Moreover, chrysin and juglanin attenuate inflammatory signaling responses by suppressing COX-2, IL-6, and TNF-α expression across multiple animal cancer models (Kong and Xu, 2020; Song K. et al., 2017).

Certain marine polysaccharides of ESCM constituents have been shown to regulate adaptive immunity. Laminarin enhances dendritic cell maturation and antigen presentation, leading to robust cytotoxic T-cell activation and reduced growth and hepatic metastasis of melanoma tumor (Park et al., 2020). Similarly, fucoidan promotes cytotoxic T-lymphocytes proliferation and cytokine production, significantly suppressing CT-26 carcinoma growth *in vivo* (Zhou et al., 2024).

The NLRP3 inflammasome serves as a double-edged sword in carcinogenesis, mediating protective immune responses while driving pathological inflammation when dysregulated (He et al., 2016). Homoplantaginin inhibits caspase-1 activation through epigenetic modulation of inflammasome components, effectively blocking IL-1β processing and inflammatory cascade amplification (Zhang et al., 2021). This effect is mechanistically distinct from galangin, which suppresses aberrant NLRP3 activation in ovarian cancer xenograft mouse models, downregulating both NLRP3 expression and IL-1β maturation (Hoesel and Schmid, 2013).

#### 3.4.2 Inhibition of the NF-κB pathway

Nuclear factor-κB (NF-κB) is a transcription factor highly associated with inflammation, mainly composed of the p65 subunit and the p50 subunit. Activation of NF-κB promotes cancer progression by inducing various genes responsible for cancer cell survival, proliferation, and metastasis, and interacts with multiple cancer-associated pathways such as PI3K/Akt, AMPK, and MAPK (Chen et al., 2020). Several constituents have been reported to inhibit the upregulation of the NF-κB pathway caused by different pathological factors. Quercetin can reduce the production of inflammatory factors such as TNF-α, COX-2, and IL-6, and inhibit TNF-α-induced apoptosis and inflammation by blocking NF-κB signaling pathway (Li et al., 2024). Homoplantaginin similarly attenuates TNF-α-induced inflammation, but it is the NF-κB/MAPK signaling pathway that is blocked (Liu et al., 2013).

Toll-like receptor 4 (TLR4) is predominantly expressed on macrophages. As an upstream factor of NF-κB, it can influence downstream transcription factors through the TLR4/NF-κB signaling pathway and drive tumor progression during chronic inflammation. Activation of TLR4 on macrophages stimulates increased secretion of the cytokines IL-10, MMP-2, and MMP-9. It not only accumulates inflammatory damage, but also increases cancer cell proliferation and migration (Zhang and Xu, 2018). Juglanin inhibits NF-κB activation induced by IL-1β and also blocks the TLR4/NF-κB pathway, significantly reducing pro-inflammatory cytokine production induced by lipopolysaccharide (LPS), and to ameliorate inflammation (Han MA. et al., 2016). Galangin likewise significantly induces apoptosis in renal cancer (Caki, ACHN and A498) cells but not normal cells by inhibiting NF-κB pathway activation, which induces downregulation of Bcl-2 protein and survivin expression at the transcriptional level (Hou et al., 2018). In the meantime, juglanin significantly attenuates both p38/JNK and PI3K/Akt signaling pathways to inhibit NF-κB activation induced by UVB *in vivo* and *in vitro* (Khan et al., 2011a). After applying chrysin to early hepatocellular carcinoma cells induced by N-nitrosodiethylamine (DEN), the expression of COX-2 and NF-κB was significantly reduced at both mRNA and protein levels, similarly, the level of the anti-apoptotic marker Bcl-xL was decreased, whereas the expression of p53, Bax, and caspase-3 was elevated, which inhibited DEN-induced hepatocellular carcinoma cell proliferation and apoptosis (Khan et al., 2011b; Batlle and Massagué, 2019).

#### 3.4.3 Inhibition of TGF-β expression

Transforming growth factor (TGF)-β is an important enforcer of immune homeostasis, associating many constituents and functions of the immune system, and perturbations in its signaling underlie the pathology of inflammatory diseases (Derynck and Zhang, 2003). Smad proteins are intracellular effectors of TGF-β signaling, activation of the TGF-β/Smad signaling pathway will promote EMT (Hu et al., 2017). The extract peptide of biejia can inhibit the TGF-β1/Smad pathway, alter the expression levels of various types of collagen in the extracellular matrix, and inhibit the activation and proliferation of the hepatic stellate cell line, HSC-T6, which was induced by TGF-β1 (Tang et al., 2013; Guo Y. et al., 2021). Quercetin antagonizes the TGF-β/Smad signaling pathway by decreasing TGF-β1 levels and can inhibit EMT, thereby inhibiting the growth, migration and invasion of pancreatic cancer cells and inducing its apoptosis (Chen QQ. et al., 2023).

#### 3.4.4 Increasing intake of amino acids

Both biejia and guijia have been shown to be rich in amino acids (Zou et al., 2022; Zhao et al., 2020). Amino acids can assist innate immunity and play an effector function in the survival and proliferation of immune cells (Wang and Zou, 2020). A variety of amino acids are involved in the regulation of immune responses in the tumor microenvironment and are involved in immunotherapy of cancer (Yang et al., 2023b). Increasing amino acid content and reconnecting amino acid metabolism can enhance immunity and treat inflammation and cancer (Jelic et al., 2021).

### 3.5 Anti-oxidant

Reactive oxygen species (ROS) damage lipids, nucleic acids, and proteins, thereby altering their function. A state of oxidative stress occurs when the balance between ROS production and ROS scavenging by anti-oxidant defenses is disturbed (Klaunig, 2018). Oxidative stress produces multiple pathological products, such as Superoxide Dismutase (SOD), Malondialdehyde (MDA), etc., which cause damage to cellular macromolecules and most importantly, mutations in genomic DNA (Blaser et al., 2016). In addition, NF-κB and ROS interact and promote each other in a positive feedback loop (Dhar et al., 2002; Guan et al., 2018). ROS mediate multiple protein expression and signaling pathways downstream, including TGF-β and EGFR/MEK/ERK pathways (Wang et al., 2018; Vermot et al., 2021). Anti-oxidants are not only anti-inflammatory but also effective in preventing DNA mutations. Physiological processes associated with resistance to oxidative stress, including inhibition of expression of nicotinamide adenine dinucleotide phosphate oxidase (NOX) protein, which is a ROS-producing enzyme, and activation of the nuclear factor erythroid 2-related factor 2 (NRF2) pathway that is resistant to oxidative damage (Figure 4) (He et al., 2020; Heo and Jeon, 2009).

It has been demonstrated that fucoxanthin can enhance the anti-oxidant capacity of the organism, increase the SOD activity, reduce the MDA content, and have the ability to resist oxidative stress (Meng et al., 2022). Homoplantaginin inhibited oxidized low-density lipoprotein (ox-LDL)-induced cellular damage through activation of the NRF2 anti-oxidant signaling pathway and reduced ROS production, ERK phosphorylation, and NF-κB transcription (Wang et al., 2021). Juglanin downregulates the expression of NOX4, decreases SOD activity, and inhibites the caspase-1 axis activation, and reduces IL-1β and IL-18 production, ameliorating cellular damage and exerting anti-inflammatory and anti-oxidant physiological utility (Sul and Ra, 2021). Quercetin has both anti-inflammatory and anti-oxidant effects. It reduces LPS-induced elevation of intracellular ROS levels and inhibits LPS-stimulated NOX2 mRNA and protein expression. It also inhibits the nuclear translocation of NF-κB and decreases the levels of IL-1 and IL-6 (Michalcova et al., 2019). Isoquercitrin inhibits the production of ROS in ovarian cancer cells, while kaempferol inhibits the production of ROS in the bone marrow-derived neutrophils of the mice mammary tumor model, thus limiting the oxidative stress (Zeng et al., 2020; Doi et al., 2021).

## 4 Clinical trials

The establishment of clinical trials for CMM is a crucial step to enhance their safety and efficacy, as well as a necessary measure to gain international recognition (He et al., 2018; Tsai et al., 2023). Meanwhile, *in vitro* and *in vivo* experiments have demonstrated the exact therapeutic effect of ESCM on cancer, and its clinical translation should be the next research focus. Randomized controlled trials have shown that as complementary treatments, fucoidan and luteolin can effectively improve the quality of life of terminal cancer patients after undergoing radiotherapy or chemotherapy (Takahashi et al., 2018; Naiki et al., 2025; Rui et al., 2023). Although clinical trial evidence proving ESCM’s therapeutic effect on cancer remains limited, there are currently ongoing clinical trials investigating this. In order to clarify the declaration of clinical trials of ESCM, we searched for relevant clinical trials both domestically and internationally on the International Clinical Trial Registry Platform (https://trialsearch.who.int/) with the keywords of ESCM, the constituents, and “cancer”. The results were listed in Table 3. It indicated that colon cancer can be prevented with rutin as a dietary supplement (NCT00003365). Luteolin can be used to treat prostate cancer (JPRN-jRCTs041230029), and inhibit ovarian cancer stem cells proliferation by interfering with the KDM4C/PPP2CA/YAP pathway (ChiCTR2200056567). Quercetin, in combination with other therapeutic modalities, is effective in the treatment of desmoplasia-resistant prostate cancer (NCT06615752), and also reverses chemotherapy resistance in triple-negative breast cancer (NCT06355037). Quercetin and its encapsulated nanoparticles can also be therapeutic for cell line of tongue squamous cell carcinoma (NCT05456022).

**TABLE 3:** Clinical trials of ESCM.

Constituent	Indications	Main ID	Public title
Rutin	Colon cancer	NCT00003365	Sulindac and Plant Compounds in Preventing Colon Cancer
Luteolin	Prostate cancer	JPRN-jRCTs041230029	Safety analysis of luteolin for prostate cancer
	Ovarian cancer	ChiCTR2200056567	Inhibition of ovarian cancer stem cells by luteolin targeting the KDM4C/PPP2CA/YAP pathway: an *in vitro* observational clinical study
Quercetin	Desmoplasia-resistant prostate cancer	NCT06615752	Green Tea and Quercetin in Combination with Docetaxel Chemotherapy in Castration-resistant Prostate Cancer Patients
	Triple-negative breast cancer	NCT06355037	Dasatinib Combined With Quercetin to Reverse Chemo Resistance in Triple Negative Breast Cancer
	Tongue squamous cell carcinoma	NCT05456022	Therapeutic Efficacy of Quercetin Versus Its Encapsulated Nanoparticle on Tongue Squamous Cell Carcinoma Cell Line

## 5 ESCM-related health products with anti-cancer effects

The creation of health products stands as a crucial application of ESCM in daily life, offering transparent ingredients and proven efficacy while being tailored to meet the needs of various health scenarios. To explore how ESCM is used in daily health products, we searched China’s State Administration for Market Regulation’s Special Food Query Platform (http://ypzsx.gsxt.gov.cn/specialfood/#/food) using “ESCM” as the search term. We found that health products mainly made from ESCM combined with other CMM ingredients have both nutritional and healthcare functions. They also have cancer prevention potential by clearing cancer risk factors (Table 4). The full details are in the Supplementary Material (Supplementary Table S1). This is specifically evident in five aspects: first, boosting immunity and strengthening immune system defenses to avoid cellular carcinogenesis induced by chronic inflammatory damage (Anbarasu and Anbarasu, 2023); Second, maintaining healthy levels of serum lipid, and improving obesity, preventing against types of cancer caused by hyperlipemia and obesity-induced abnormal hormone levels (Boguszewski and Boguszewski, 2019; Radišauskas et al., 2016); Third, maintaining healthy levels of blood pressure, preventing against the kidney cancer, prostate cancer, and colon and rectal cancer, that are highly associated with hypertension (Duan et al., 2014); Fourth, maintaining healthy levels of glucose, preventing against the apoptosis resistance in cancer cells and hyper-inflammation highly associated with hyperglycemia (Ghar et al., 2024; Han H. et al., 2020); Fifth, decreasing chemical substance-induced liver damage, and protecting against the hepatotoxicity of chemical products, thereby reducing the incidence of liver cancer (Baell, 2010).

**TABLE 4:** ESCM-related health products with anti-cancer effect**s**.

Efficiency	ESCM	Product name	Principal raw material	License number
Enhancement of immunity	Biejia	Tangyuan Brand Ginseng, Wolfberry and Turtle Capsule	Lycii Frustus, Poria, Dioscoreae Rhizoma, Trionycis Carapax, Ginseng Radix et Rhizoma extract	G20090465
	Guijia	Songyou Drink Brand Danqi Goujia Granules	Lycii Frustus, Hawthorn, Testudinis Carapax Et Plastrum, Astragali Radix, Salviae Miltiorrhizae Radix Et Rhizoma	G20070160
		Leping Brand Kamehameha Boneset Capsules	Astragali Radix extract, Eucommiae Cortex extract, Lycii Frustus extract, Testudinis Carapax Et Plastrum extract, Drynariae Rhizoma extract, Calcium carbonate, Magnesium stearate	G20190045
		Huiren Brand Rehmannia Glutinosa Tablets	Rehmanniae Radix Praeparata, Testudinis Carapax Et Plastrum, Ligustri Lucidi Fructus, Rhodiolae Crenulatae Radix Et Rhizoma, Alpiniae Oxyphyllae Fructus, Fructus Mori, Gardeniae Fructus	G20190386
	Guijia, Lurong	Suhang Brand Antler and Turtle Wine	Cervi Cornu Pantotrichum, Testudinis Carapax Et Plastrum, Astragali Radix, Poria, Lycii Frustus, White spirits	G20030041
		Yellow Gold Medal Deer Antler, Kamehameha and American Ginseng Wine	Cervi Cornu Pantotrichum, Testudinis Carapax Et Plastrum, Panacis Quinquefolii Radix, Eucommiae Cortex, Lycii Frustus, Honey, White spirits, Water	G20060057
		Hongjitang Brand Ginseng, Deer Antler and Turtle Wine	Morindae Officinalis Radix, Lycii Frustus, Fructus Mori, Alpiniae Oxyphyllae Fructus, Ginseng Radix Et Rhizoma, Testudinis Carapax Et Plastrum, Cervi Cornu Pantotrichum, Cinnamomi Cortex	G20130530
		Weixiong Brand Horse Deer Antler and Western Ginseng Wine	Testudinis Carapax Et Plastrum, Acanthopanacis Senticosi Radix Et Rhizoma Seu Caulis, Lycii Frustus, Longan Arillus, Cervi Cornu Pantotrichum, Panacis Quinquefolii Radix	G20220341
	Haizao	Foucauld’s Seaweed Oral Liquid	Sargassum, Honey, Citric acid, Benzoic Acid, Stevioside, Drinking water	J20060005
		Bioscan Brand Chitosan Algae Yam Capsules	Dioscoreae Rhizoma, Sargassum, Chitosan	G20110403
	Lurong^a^	Lutetan Chewable Antler and Western Ginseng Tablets	Cervi Cornu Pantotrichum, Panacis Quinquefolii Radix, Epimedii Folium, Ophiopogonis Radix, Mannitol, Aspartame (with L-Phenylalanine), Chocolate-Ice cream flavoring, Creamy flavoring	G20100153
		FangZhongFang Brand Beef Knee and Deer Antler Wine	Cervi Cornu Pantotrichum, Rehmanniae Radix, Morindae Officinalis Radix, Achyranthis Bidentytae Radix, Lycii Frustus, Poria, Hippophae Fructus, White spirits, Water	G20110596
		Shurentang Brand Sanqi Xiyangshen Ma Deer Antler Wine	Cervi Cornu Pantotrichum, Notoginseng Radix Et Rhizoma, Panacis Quinquefolii Radix, Ganoderma, Polygonati Rhizoma, Lycii Frustus, Hippophae Fructus, Fructus Mori	G20110717
		Zhangzisong Brand Antler Maidenhair Wine	Cervi Cornu Pantotrichum, Angelicae Sinensis Radix, Lycii Frustus, Ligustri Lucidi Fructus, Crystal sugar, White spirits, Purified water	G20130403
		Tongrentang Brand Horsetail Antler and Western Ginseng Capsules	Cervi Cornu Pantotrichum, Lycii Frustus extract, Ophiopogonis Radix extract, Ganoderma extract, Panacis Quinquefolii Radix extract	G20140691
		President Brand Ganoderma Lucidum Antler Tablets	Cervi Cornu Pantotrichum (irradiated), Lycii Frustus extract, Ophiopogonis Radix extract, Ganoderma extract, Panacis Quinquefolii Radix extract	G20141048
		Aoqi Long Brand Xiyangshen Deer Antler Wine	Astragali Radix, Hippophae Fructus, Lycii Frustus, Polygonati Rhizoma, Panacis Quinquefolii Radix, Cervi Cornu Pantotrichum	G20141109
		Luyuanchun Brand Horse Deer Antler Soft Capsules	Cervi Cornu Pantotrichum powder (irradiated)	G20160063
		Overseas Brand Ma Deer Antler and Western Ginseng Soft Capsules	Cervi Cornu Pantotrichum powder, Panacis Quinquefolii Radix extract, Soybean oil, Beeswax, Gelatin, Glycerol, purified water, Titanium dioxide	G20160427
		Ausflex Brand Ginseng and Antler Capsules	Cervi Cornu Pantotrichum powder (irradiated), Ginseng Radix Et Rhizoma extract	G20220183
		Zhonghe Hongye Brand Horse Deer Antler and Western Ginseng Tablets	Cervi Cornu Pantotrichum powder (irradiated), Panacis Quinquefolii Radix extract	G20220217
		Healthy Deer Sang Brand Ma Deer Antler Tablets	Cervi Cornu Pantotrichum (irradiated)	G20230116
		Royal Shop Brand Xiyangshen Ma Deer Antler Wine	Cervi Cornu Pantotrichum, Panacis Quinquefolii Radix, White spirits, purified water	G20230151
		Ruiyuan Brand Cistanche Antler Capsules	Cervi Cornu Pantotrichum powder, Cordyceps militaris mycelium powder, Cistanches Herba extract, Magnesium stearate, Magnesium stearate	G20230177
		Jiyun Brand Antler Powder Capsules	Cervi Cornu Pantotrichum powder	G20230557
		Herbal Essence Zhengyuantang Brand Horse Deer Antler and American Ginseng Tablets	Cervi Cornu Pantotrichum powder (irradiated), Panacis Quinquefolii Radix extract, Microcrystalline cellulose, Maltodextrin, Carboxymethylstach sodium, Magnesium stearate	G20230717
	Muli	Haiwang Brand Golden Oyster Capsules	Ostreae Concha powder, Amylum	G20040491
		Hongyangshen Brand Chitosan Oyster Tablets	Chitosan, Ostreae Concha extract	G20141320
		Golden Olive Brand Oyster Taurine Vitamin C Capsules	Ostreae Concha extract, Taurine, Vitamin C	G20150237
		Kemper Brand Sea Cucumber Oyster Capsules	Sea cucumber extract, Ostreae Concha extract	G20160459
		Chinese Salamander Brand Marine Fish Oligopeptide Oyster Oral Liquid	Marine fish oligopeptide powder, Ostreae Concha extract, Blueberry extract, Citric acid, Pectin, Sucralose, Ethyl Maltol, Purified water	G20200193
		Xuelong Brand Oyster Capsules with Sea Cucumber	Ostreae Concha extract, Sea cucumber powder, Lycii Frustus extract	G20230110
	Xuanshen	Ruinian Brand Xuan Ginseng and Western Ginseng Amino Acid Capsules	Silkworm pupa composite amino acid powder, Scrophulariae Radix extract, Panacis Quinquefolii Radix extract	G20141001
		Hop Fai Brand Xiyangshen Xuan Shen Sheng Di Huang Granules	Scrophulariae Radix extract, Rehmanniae Radix extract, Ophiopogonis Radix extract, Panacis Quinquefolii Radix extract, Dextrin, Stevia sugar	G20230352
		Xinzhu Nutritional Brand Di Huang Xuan Shen Tablets	Rehmanniae Radix, Scrophulariae Radix, Fritillariae Thunbergii Bulbus, Moutan Cortex, Menthae Haplocalycis Herba, Ophiopogonis Radix, Glycyrrhizae Radix Et Rhizoma, White granulated sugar, Povidone K 30, Carboxymethylstach Sodium, Magnesium stearate	G20230698
		Hop Fai Brand Xiyangshen Xuan Ginseng Sheng Di Huang Tablet	Scrophulariae Radix extract, Rehmanniae Radix extract, Ophiopogonis Radix extract, Panacis Quinquefolii Radix extract, Microcrystalline Cellulose, Magnesium stearate	G20230703
		Hop Fai Brand Xiyangshen Xuan Ginseng Sheng Di Huang Capsules	Scrophulariae Radix extract, Rehmanniae Radix extract, Ophiopogonis Radix extract, Panacis Quinquefolii Radix extract, Microcrystalline Cellulose	G20230704
Maintaining healthy levels of serum lipid, and improving obesity	Juemingzi^a^	Baibang Brand Lotus Leaf Cassia Seed Capsules	Cassiae Semen, Tea polyphenols, Ginkgo Folium, Nelumbinis Folium, Amylum	G20040410
		Zhongyantong Brand Cassia Seed Gynostemma Tablets	Cassiae Semen, Gynostemma pentaphyllum Makino	G20050050
		Green Slim Brand Cassia Seed and Lotus Leaf Capsules	Cassiae Semen, Coicis Semen, Alismatis Rhizoma, Poria, Nelumbinis Folium, Puerariae Lobatae Radix	G20050898
		Kangjiafu Brand Cassia Seed Ginkgo Biloba L-Carnitine Capsules	Cassiae Semen, Nelumbinis Folium, Poria, Alismatis Rhizoma, Ginkgo Folium, Wheat germ powder, L-carnitine	G20060376
		Qianquan Brand Cassia Seed Zelda Lotus Leaf Tea	Cassiae Semen, Alismatis Rhizoma, Oolong tea (irradiated), Nelumbinis Folium (irradiated), Gynostemma pentaphyllum Makino	G20120004
		Viridian Brand Cassia Seed and Lotus Leaf Capsules	Cassiae Semen, Ginkgo Folium, Alismatis Rhizoma, Gynostemma pentaphyllum Makino, Nelumbinis Folium	G20130046
		Fuzhen Brand L-Carnitine Tea Polyphenol Cassia Seed and Lotus Leaf Capsules	Cassiae Semen extract, Nelumbinis Folium extract, Tea polyphenols, L-carnitine, Magnesium stearate	G20141178
		Hongyangshen Brand Lotus Leaf Zelda Cassia Seed Extract Tablets	Cassiae Semen extract, Nelumbinis Folium extract, Alismatis Rhizoma extract, Oolong tea extract	G20150208
		St. Neve’s Cassia Seed and Lotus Leaf Tea	Cassiae Semen, Nelumbinis Folium, Puer tea, Alismatis Rhizoma, Gynostemma pentaphyllum Makino, Siraitiae Fructus	G20160083
		Tianzhiyuan Brand Cassia Seed Gynostemma Oral Liquid	Cassiae Semen, Puerariae Lobatae Radix, Hawthorn, Gynostemma pentaphyllum Makino, Nelumbinis Folium, Alismatis Rhizoma	G20210065
		River Rain Brand Cassia Seed Poria Beverage	Cassiae Semen extract (irradiated), Poria extract (irradiated), Alismatis Rhizoma extract (irradiated), Nelumbinis Folium extract (irradiated), Apple cider vinegar, Concentrated apple juice, Concentrated hawthorn juice, Honey, Sucralose, Edible flavorings, Purified water	G20230886
		Yishuitan Brand L-Carnitine Cassia Seed Capsules	Cassiae Semen, Gynostemma pentaphyllum Makino, L-Carnitine-L-Tartrate, Nelumbinis Folium, Microcrystalline cellulose, Magnesium stearate	G20240313
		Runxintang Carnitine Cassia Seed Tablets	Cassiae Semen extract, L-Carnitine-L-Tartrate, Alismatis Rhizoma extract, Nelumbinis Folium extract, Astragali Radix extract, Dextrin, Magnesium stearate	G20240320
		Green Slim Brand Cassia Seed Lotus Leaf Zelda Drink	Cassiae Semen, Nelumbinis Folium, Alismatis Rhizoma, Potato extract, Citric acid, Citrus reticulata Blanco extract, orange flavoring, Sucralose, Purified water	G20240361
	Kunbu, Juemingzi	Haiku Brand Cassia L-Carnitine Capsules	Cassiae Semen, Poria, Laminariae Thallus, Chitosan, L-carnitine, Amorphophallus konjac powder, Spirulina powder, Margarita	G20070249
		Jinjiaoli Brand Lotus Leaf Cassia Seed and Polygonum Multiflorum Capsules	Polygoni Multiflori Radix extract, Nelumbinis Folium extract, Cassiae Semen extract, Alismatis Rhizoma extract, Laminariae Thallus extract	G20100283
		Huanghong Brand Perilla Seed Cassia Seed Angelica Dahurica Capsules	Perillae Fructus, Cassiae Semen, Angelicae Dahuricae Radix, Laminariae Thallus, Coicis Semen	G20100547
	Lurong	Zhangzisong Brand Antler and Ginseng Oral Liquid	Ginkgo Folium, Hawthorn, Alismatis Rhizoma, Ginseng Radix Et Rhizoma, Cervi Cornu Pantotrichum	G20140455
Maintaining healthy levels of blood pressure	Juemingzi	Jinnuo Tong Brand Astragalus Danshen Cassia Seed Capsules	Astragali Radix, Salviae Miltiorrhizae Radix Et Rhizoma, Ginkgo Folium, Puerariae Lobatae Radix, Cassiae Semen, Chrysanthemi Flos, Hippophae Fructus	G20100311
		Tian Keng Brand Rhizoma Pinelliae Cassiae Cortex Eucommiae Capsules	Apocyni Veneti Folium, Eucommiae Cortex, Cassiae Semen, Hawthorn	G20120667
		Jinnuo Tong Brand Astragalus Danshen Cassia Seed Capsules	Astragali Radix, Salviae Miltiorrhizae Radix Et Rhizoma, Ginkgo Folium, Puerariae Lobatae Radix, Cassiae Semen, Chrysanthemi Flos, Hippophae Fructus	G20240224
Maintaining healthy levels of glucose	Haizao	Fumitra Brand Sodium Alginate Chromium Yeast Powder	Sodium alginate, Calcium chloride, Chromium yeast, Sodium citrate, Silicon dioxide	G20240134
	Juemingzi	Hong Kong Sang Brand Ginseng Cassia Seed Yellow Essence Oral Liquid	Ginseng Radix Et Rhizoma, Schisandrae Chinensis Fructus, Cassiae Semen, Ligustri Lucidi Fructus, Salviae Miltiorrhizae Radix Et Rhizoma, Polygonati Rhizoma, Lycii Frustus, Puerariae Lobatae Radix, Jujubae Fructus	G20080612
	Lurong	Camphor Pine Brand Deer Antler Maitake Oral Liquid	Astragali Radix, Rehmanniae Radix Praeparata, Ophiopogonis Radix, Dioscoreae Rhizoma, Cervi Cornu Pantotrichum	G20140566
	Xuanshen	Tongrentang Brand Astragalus and Ginseng Tea	Mori Cortex, Fructus Mori, Astragali Radix, Mori Folium, Natrii Sulfas Exsiccatus, Chromium Picolinate	G20130058
Auxiliary protection from chemical liver injury	Haizao	Blue Key Brand Sodium Alginate Taurine Capsules	Sodium alginate (irradiated), Taurine (irradiated)	G20070380
	Juemingzi	Herbalife Brand Cassia Seed Panax ginseng and Fructus Schisandrae chinensis Capsules	Cassiae Semen extract, Notoginseng Radix Et Rhizoma extract, Panacis Quinquefolii Radix extract, Schisandrae Chinensis Fructus extract	G20110448
		Yipintang Brand Cassia Seed, Green Peel and Pericarp Tea	Cassiae Semen, Citri Reticulatae Pericarpium, Citri Reticulatae Pericarpium Viride	G20240309
	Muli	Haiwang Jintu Brand Oyster Soy Peptide Carnitine Oral Liquid	Purified water, Ostreae Concha extract, Soybean peptide powder, L-carnitine, Taurine, Zinc gluconate, Vitamin B6, Vitamin C, Fructose syrup, Honey, Soluble dietary fiber, Beta-cyclodextrin, Peppermint flavoring	G20110347
		Shanggong Qiangshengtang Brand Oyster, Pueraria dulcis and Hovenia dulcis Capsules	Puerariae Lobatae Radix extract, Auranth Fructus Immaturus extract, Schisandrae Chinensis Fructus extract, Ostreae Concha powder, Tea polyphenols, Microcrystalline cellulose	G20190159

^a^ The number of healthy products in this column exceeds 30, thereby, only products with the ESCM, as the top one of the ingredient list are retained.

## 6 Conclusion

As a part of the primary prevention against cancer, the application of ESCM is of great importance. This paper comprehensively analyzes the research progress of ESCM in the field of anti-cancer applications, and provides insights into the multiple anti-cancer mechanisms of the active constituents in these herbs. The findings indicate that these constituents have significant inhibitory effects on the growth, survival and metastasis of cancer cells through various biological pathways. Specifically, the active constituents were found to inhibit cancer cell growth and metastasis by affecting mitochondrial function, regulating the expression of apoptosis-related proteins, and interfering with cell signaling pathways such as PI3K/AKT, AMPK, MAPK, and EGFR. They also induced cell cycle arrest by modulating the expression of cell cycle proteins and cell cycle-dependent kinases. Additionally, these constituents reduced the invasiveness and metastatic ability of cancer cells by down-regulating MMP expression and inhibiting the EMT process. They decreased the vascular supply to tumors by reducing VEGF expression and affecting VEGF-mediated signaling pathways. Furthermore, the active constituents were shown to reduce inflammatory responses and oxidative stress by inhibiting the release of inflammatory factors, blocking the NF-κB signaling pathway, and suppressing TGF-β expression. They also enhanced the activity of amino acids and antioxidant enzymes to regulate immune responses and inflammatory states. Beyond biological experiments, clinical trials and the application of health products have provided further evidence of ESCM’s anti-cancer effects.

Despite the broad-spectrum anti-cancer properties of the reviewed ESCM constituents, it is noteworthy that certain constituents in Figure 1 (e.g., quercetin, kaempferol, rutin) represent well-documented pan-assay interference compounds (PAINS) (Magkoufopoulou et al., 2011; Wang et al., 2017; Gao et al., 2021; Jomova and Valko, 2011). These chemical scaffolds frequently yield screening hits due to nonspecific interfering mechanisms such as redox cycling that can generate reactive oxygen species or covalent adducts and metal chelation that can disrupt metalloenzyme function, leading to false-positive results across diverse assay formats (Hermann et al., 2012; Xie et al., 2011). Additionally, small molecules often exhibit multiple unintended biological targets and such off-target effects may lead to preclinical and even clinical toxicities (Gobet et al., 2014). While the presence of such constituents does not inherently invalidate the academic findings, additional rigorous orthogonal validation is essential to confirm their biological relevance (Brown and Koropatkin, 2023).

To mitigate the risk of false positives in preclinical drug discovery, several practices are recommended. These include biophysical assays such as surface plasmon resonance and isothermal titration calorimetry to differentiate specific binding from nonspecific aggregation (Cheng and Lai, 2003); dose-response analyses to establish correlations between compound activity and biological relevance (Kenakin, 2019); and structure–activity relationship studies to ensure logical and consistent activity trends (Rasmussen et al., 2011; Stork et al., 2019). Apart from these experimental strategies, deep/machine learning based computational framework have been developed to predict the PAINS-like behavior and off-target interactions (Ja et al., 2018; Sherkatghanad et al., 2023; Rao et al., 2023; Hu et al., 2023). While these tools are effective for early screening, over-reliance on these filters risks discarding potentially valid leads. In the context of ESCM extract, the therapeutic effect may result from the synergistic activity of the constituents, rather than from a single highly bioactive compound (Escandón-Rivera et al., 2020; Bray et al., 2024). This highlights the importance of evaluating such extracts within a systems pharmacology framework rather than applying conventional single-compound screening paradigms. Together, an integrative strategy that combines rigorous orthogonal validation, advanced computational prediction, and systems pharmacology is essential for minimizing false-positive results and accurately evaluating the therapeutic relevance of both small molecules and complex botanical extracts of ESCM.

Given that the vast majority of the evidence in this study is from *in vitro* or *in vivo* experiments, limitations are unavoidable. Firstly, *in vitro* models lack the complexity of the human tumor microenvironment and may not fully recapitulate human cancer biology, limiting the predictive value of preclinical outcomes; secondly, many of the studies were administered at supraphysiological concentrations or via non-oral routes, which may not reflect achievable human plasma levels or feasible methods of administration, and is also a test for drug safety; and thirdlyly, most of the studies targeted isolated pathway (e.g., PI3K/Akt), whereas cancer progression involves dynamic crosstalk between multiple signaling pathway networks, and single-target interventions may lack clinical relevance. As research into the anti-cancer mechanisms of ESCM advances, our comprehension of their potential in cancer prevention and therapy continues to expand. The future research and application prospects can be envisioned in the following aspects.

1. Mechanistic exploration: Although current studies have extensively explored the anti-tumor potential of ESCM, most research remains limited to preliminary observations. Future investigations should integrate cutting-edge approaches (e.g., single-cell RNA sequencing, spatial transcriptomics) with dynamic molecular mechanism analyses to systematically unravel the molecular interaction networks and signaling pathways underlying ESCM efficacy. The in-depth exploration of mechanisms would not only facilitate the understanding of synergic effects of components in ESCM and but also enable the guidance to precision clinical applications. These advancements would ultimately bridge traditional Chinese theories with complex herbal medicine, creating a conceptual continuum between empirical knowledge and evidence-based therapeutic innovation.
2. Clinical safety and efficacy validation: Clinical safety and efficacy verification is the primary measure for the clinical translation of ESCM. To achieve this objective, critical research priorities in subsequent clinical trials include: standardization of ESCM extracts, determination of safe dosage ranges, the differences in bioavailability of each constituent and their corresponding combination methods, as well as the development of personalized therapeutic regimens stratified by cancer subtypes.
3. Combination therapy, preventive therapy and personalization: After the clinical safety and efficacy validation, the extensive clinical application of ESCM can be actualized in steps. Firstly, combination therapy: The synergistic effects of combining these ESCM with conventional treatments (e.g., chemotherapy, radiotherapy, immunotherapy) should be investigated to enhance therapeutic efficacy and mitigate side effects. Secondly, preventive treatment: Using technological means to identify high-risk cancer patients, selecting appropriate ESCM for preventive medication based on pathological changes and indications. Thirdly, personalization: Given that individuals may respond differently to the same treatment, future studies should explore how to tailor the use of ESCM for prevention and treatment based on individual genetic backgrounds and lifestyle habits.
4. Long-term studies: Long-term follow-up studies are essential to evaluate the sustained impact of ESCM use on human health and to identify any potential long-term side effects or toxicity.
5. Multidisciplinary collaboration: Dietary prevention strategies require collaborative efforts across various disciplines. Encouraging cooperation among experts in biology, pharmacy, nutrition, and modern medicine will accelerate the research and application of these ESCM in cancer prevention.

In summary, ESCM holds significant promise in the field of cancer prevention and treatment. Future research must be conducted across these multiple dimensions to fully harness the potential of these natural resources in the fight against cancer.
