# Supplementary material for: Research progress on the anti-cancer mechanisms of edible salty-flavored Chinese materia medica
Source: Front Pharmacol. 2025 Jun 20;16:1598978. doi: 10.3389/fphar.2025.1598978 (PMC12226498; doi:10.3389/fphar.2025.1598978)
Supplement: Supplementary file 1 [file Table1.DOCX]

### ***Supplementary Materials***

### **Research Progress on the Anti-cancer Mechanisms of Edible Salty-flavored Chinese Materia Medica**

Zhongqi Shen ^1#^, Meng Yu ^2#^, Zhenguo Wang ^1*^

**Affiliations**

^1^ Institute of Chinese Medical Literature and Culture, Shandong University of Traditional Chinese Medicine, Jinan, Shandong, P. R. China 250355.

^2^ Innovative Institute of Chinese Medicine and Pharmacy, Shandong University of Traditional Chinese Medicine, Jinan, Shandong, P. R. China 250355.

^*^ Correspondence should be addressed to Zhenguo Wang: zhenguow@126.com.

^#^ These authors contributed equally to this work.

### **Supplementary Materials**

Table S1. Full details of ESCM-related health products with anti-cancer effects.

***Supplementary Tables***

| **Table S1.** **Full details of** **ESCM-related health products with anti-cancer effects** | | | | |
| --- | --- | --- | --- | --- |
| Efficiency |  | Product name | Principal raw material | License number |
| Enhancement of immunity | Biejia | Tangyuan Brand Ginseng, Wolfberry & Turtle Capsule | Lycii Frustus, Poria, Dioscoreae Rhizoma, Trionycis Carapax, Ginseng Radix et Rhizoma extract | G20090465 |
|  | Guijia | Songyou Drink Brand Danqi Goujia Granules | Lycii Frustus, Hawthorn, Testudinis Carapax Et Plastrum, Astragali Radix, Salviae Miltiorrhizae Radix Et Rhizoma | G20070160 |
|  |  | Leping Brand Kamehameha Boneset Capsules | Astragali Radix extract, Eucommiae Cortex extract, Lycii Frustus extract, Testudinis Carapax Et Plastrum extract, Drynariae Rhizoma extract, Calcium carbonate, Magnesium stearate | G20190045 |
|  |  | Huiren Brand Rehmannia Glutinosa Tablets | Rehmanniae Radix Praeparata, Testudinis Carapax Et Plastrum, Ligustri Lucidi Fructus, Rhodiolae Crenulatae Radix Et Rhizoma, Alpiniae Oxyphyllae Fructus, Fructus Mori, Gardeniae Fructus | G20190386 |
|  | Guijia, Lurong | Suhang Brand Antler and Turtle Wine | Cervi Cornu Pantotrichum, Testudinis Carapax Et Plastrum, Astragali Radix, Poria, Lycii Frustus, White spirits | G20030041 |
|  |  | Yellow Gold Medal Deer Antler, Kamehameha and American Ginseng Wine | Cervi Cornu Pantotrichum, Testudinis Carapax Et Plastrum, Panacis Quinquefolii Radix, Eucommiae Cortex, Lycii Frustus, Honey, White spirits, Water | G20060057 |
|  |  | Hongjitang Brand Ginseng, Deer Antler and Turtle Wine | Morindae Officinalis Radix, Lycii Frustus, Fructus Mori, Alpiniae Oxyphyllae Fructus, Ginseng Radix Et Rhizoma, Testudinis Carapax Et Plastrum, Cervi Cornu Pantotrichum, Cinnamomi Cortex | G20130530 |
|  |  | Weixiong Brand Horse Deer Antler and Western Ginseng Wine | Testudinis Carapax Et Plastrum, Acanthopanacis Senticosi Radix Et Rhizoma Seu Caulis, Lycii Frustus, Longan Arillus, Cervi Cornu Pantotrichum, Panacis Quinquefolii Radix | G20220341 |
|  | Haizao | Foucauld's Seaweed Oral Liquid | Sargassum, Honey, Citric acid, Benzoic Acid, Stevioside, Drinking water | J20060005 |
|  |  | Bioscan Brand Chitosan Algae Yam Capsules | Dioscoreae Rhizoma, Sargassum, Chitosan | G20110403 |
|  | Lurong * | Red Brand Turtle Shell Deer Antler Amino Acid Oral Liquid | Bone marrow, goji berry, yellow essence, chasteberry, tortoise shell, complex amino acid powder, horse antler velvet | G20050840 |
|  |  | Yinling brand glucosamine deer antler powder capsules | D-glucosamine hydrochloride, red deer bone powder, goji berry extract, epimedium extract, horse antler powder | G20060205 |
|  |  | Changhua Brand Deer Antler and Tail Royal Jelly Oral Liquid | Bee Balm, Royal Jelly, Horse antler, Lime Honey, Sodium Citrate, Sorbic Acid, Edible Alcohol, Horsetail, Citric Acid | G20060796 |
|  |  | Changbai Song brand Wujia Deer Antler Blood Oral Liquid | Acanthopanax Senticosus, Equisetum Versicolor, Equisetum Versicolor Blood Powder, Fructus Lycium barbarum, Sugar, Ethanol, Sodium Citrate, Sorbic Acid, Tangerine Flavoring, Purified Water | G20070038 |
|  |  | Luyuanchun brand deer antler and American ginseng wine | American ginseng, horse antler (irradiated) | G20070161 |
|  |  | Qingren brand deer antler ginseng wine | Yellow essence, goji berries, horse antler, ginseng, cloves, sand nuts | G20070219 |
|  |  | Yuhe brand ginseng deer antler wine | Goji Berry, Epimedium, Horse antler, Ginseng, Clove, Saxifrage | G20080500 |
|  |  | Zirui Jintang brand ginseng, Ganoderma lucidum and deer antler capsules | Rhizoma Polygonati Odorati Extract, Chinese Yam Extract, Ginseng Extract, Mangrove Antler, Ganoderma Lucidum Extract, Propolis Powder (Propolis, Starch) | G20080551 |
|  |  | Happiness Comes Brand American Ginseng Deer Antler Tablets | American ginseng, horse antler | G20090308 |
|  |  | Bomiao's brand Epimedium Ginseng Deer Antler Capsules | Ginseng Powder, Yellow Ginseng Extract, Goji Berry Extract, Malus Vulgaris Powder, Epimedium Extract | G20090446 |
|  |  | Lutedan brand deer antler and American ginseng chewable tablets | Deer antler, Cynomolgus, Epimedium, Ophiopogon, Mannitol, Aspartame (contains phenylalanine), Chocolate flavor, Cream flavor | G20100153 |
|  |  | Qingniu brand ginseng deer antler wine | Ginseng, antler of Majus Vulgaris, Prepared Polygonum Multiflorum, Fructus Lycium Chinense, Mulberry, White Wine | G20100549 |
|  |  | Fenglingsen brand deer antler and American ginseng capsules | American ginseng, horse antler | G20100710 |
|  |  | Runxintang ass hide glue deer antler granule | Poria cocos, yam, Angelica sinensis, jujube, Colla Corii Asini, Mamaku antler | G20110124 |
|  |  | Yongde Xing brand American ginseng and deer antler capsules | American ginseng, antler velvet, starch, magnesium stearate | G20110271 |
|  |  | Global brand American ginseng, astragalus, deer antler wine | American ginseng, mammon dong, astragalus, antler velvet, Chinese yam, goji berries, white wine, rock sugar, water. | G20110516 |
|  |  | Fangfangfang brand Achyranthes and Deer Antler Wine | Velvet antler, Radix et Rhizoma Dioscoreae, Morinda Officinalis, Radix Achyranthis Bidentatae, Radix Achyranthis Bidentatae, Fructus Lycium Chinense, Poria Cocos, Sea buckthorn, White Wine, Water | G20110596 |
|  |  | Weilekang brand royal jelly, American ginseng and deer antler capsules | American ginseng extract, royal jelly lyophilized powder, goji berry extract, horse antler powder, acanthopanax senticosus extract, starch, magnesium stearate | G20110654 |
|  |  | Shurentang brand Sanqi American ginseng and deer antler wine | Velvet antler, Panax notoginseng, Panax ginseng, Ganoderma lucidum, Rhizoma Polygonati Odorati, Fructus Lycium Chinense, Sea buckthorn, Mulberry | G20110717 |
|  |  | Fangfangfang brand Achyranthes and Deer Antler Capsules | Achyranthes bidentata, Pueraria lobata, Radix Rehmanniae Praeparata, Epimedium brevicornum, Antler velvet, starch, magnesium stearate. | G20120550 |
|  |  | Miao Girl brand ginseng, clam, scale, horse deer antler wine | Jujubes, goji berries, yellow essence, mealybugs, ginseng, horse antlers | G20130009 |
|  |  | Duomeijiao Ginseng Deer Antler ass hide glue Granules | Angelica sinensis, Hawthorn, Fructus Lycium barbarum, Colla Corii Asini, Ginseng, Equisetum Versicolor, Hemoglobin Iron | G20130014 |
|  |  | Baolikang brand royal jelly, Danshen deer antler capsules | Royal Jelly Freeze-Dried Powder, Salvia Miltiorrhiza Extract, Poria Cocos Extract, Antler Powder, Silver Fungus Extract, Radix et Rhizoma Ginseng Extract | G20130105 |
|  |  | Zhangzisong brand deer antler and privet wine | Velvet horse antler, angelica, goji berries, chasteberry, rock sugar, white wine, purified water | G20130403 |
|  |  | Songhe Wenyuan brand deer antler and American ginseng wine | Chrysanthemum Cordyceps, Radix Rehmanniae Praeparata, Chinese yam, Hawthorn, Pueraria Mirifica, Lily of the Valley, Semen Coix lacryma, Cynanchum officinale, Malus antler, Cinnamon. | G20130456 |
|  |  | Yulu brand ginseng deer antler wine | Astragalus, Fructus Lycium barbarum, Ginseng, Equisetum vulgare, Clove, Serratula officinalis | G20130666 |
|  |  | Zhonglu Brand Western Ginseng and Deer Antler Oral Liquid | Goji Berry, Rhodiola Rosea, Radix et Rhizoma Ginseng, Mamaku Antler Powder | G20140690 |
|  |  | Tongrentang brand deer antler and American ginseng capsules | Antler velvet, Fructus Lycium barbarum extract, Ophiopogon extract, Ganoderma lucidum extract, Radix et Rhizoma Ginseng Extract | G20140691 |
|  |  | Runxintang brand Epimedium Ginseng Deer Antler Capsules | Ginseng (irradiated), Goji Berry, Epimedium, Horsetail Antler Powder (irradiated) | G20140731 |
|  |  | Yijia brand spore powder deer antler capsules | Penicillium mycelium powder, wall-broken Ganoderma lucidum spore powder (irradiated), Agaricus blazei mushroom extract, Ashwagandha flower extract, Equisetum vulgare (irradiated) | G20141038 |
|  |  | Presidential brand Ganoderma lucidum and deer antler slices | Antler (irradiated), Lycium barbarum extract, Ophiopogon extract, Ganoderma lucidum extract, Radix et Rhizoma Ginseng extract | G20141048 |
|  |  | Ruizhitang brand Lingzhi Gejie Ma Deer Antler Goji Berry Wine | Ganoderma lucidum, mealybugs, horse antler, leek seeds, goji berries, white wine | G20141098 |
|  |  | Aoqilong brand American ginseng deer antler wine | Astragalus, Sea buckthorn, Goji barbarum, Yellow essence, Cynoginseng, Malus antler | G20141109 |
|  |  | Su Jing brand ginseng and deer antler capsules | Penicillium chrysogenum mycelium, Horse antler, Ginseng extract | G20141149 |
|  |  | Dangxiangju brand ginseng and Epimedium wine | Astragalus, Astragalus membranaceus, Fructus Lycium barbarum, Epimedium brevicornum, Ginseng, Equisetum vulgare | G20150053 |
|  |  | Jiaoshengtang ass hide glue ginseng antler oral liquid | Goji Berry, Prepared Di Huang, Colla Corii Asini, Ginseng, Ma Deer Antler | G20150331 |
|  |  | Banlong brand deer antler and ginseng capsules | Maitake, Horse antler, Ginseng | G20150599 |
|  |  | Honor brand sea cucumber, wolfberry, deer antler slices | Sea cucumber powder (irradiated), Fructus Lycium barbarum extract, Malus antler powder (irradiated) | G20150896 |
|  |  | Qingchen brand American ginseng, deer antler, yam capsules | Goji berries, yam, Panax ginseng (irradiated), horse antler powder (irradiated) | G20150934 |
|  |  | Jinpai Cordyceps, Ginseng, Horse Deer Antler, Iron Skin, Dendrobium, and Ligustrum Capsules (Men's Type) | Cordyceps Sinensis, antler velvet, ginseng, dendrobium, chasteberry, silicon dioxide, lactose, magnesium stearate | G20160046 |
|  |  | Luyuanchun brand velvet antler soft capsules | Horse antler powder (irradiated) | G20160063 |
|  |  | Jianbol Danggui Deer Antler Soft Capsules | Angelica sinensis, Raspberry, Morinda citrifolia, Horse antlers | G20160108 |
|  |  | Tianyitang brand deer antler dendrobium slices | Ginseng, Fructus Lycium barbarum, Dendrobium officinale, Malus antler powder | G20160257 |
|  |  | Northeast Deer Ace Maca American Ginseng and Deer Antler Wine | Maca Powder, Rhizoma Polygonati Odorati, Astragalus Membranaceus, Fructus Lycium barbarum, Ophiopogon, Panax Ginseng, Equisetum vulgare | G20160312 |
|  |  | Overseas brand deer antler and American ginseng soft capsules | Antler Powder, Cucumber Extract, Soybean Oil, Beeswax, Gelatin, Glycerin, Purified Water, Milk Chocolate Brown (Bright Blue, Sunset Yellow, Carmine Red), Titanium Dioxide | G20160427 |
|  |  | Shenghao brand American ginseng, Rhodiola rosea, and deer antler capsules | Radix Panax Ginseng, Acanthopanax Senticosus, Rhodiola Rosea, Rhizoma Polygonati Odorati, Poria Cocos, Mulberry, Jujubae, Malus Vulgaris, Dextrin, Magnesium Stearate | G20160454 |
|  |  | Golden Life Brand Deer Antler and American Ginseng Capsules | Royal Jelly Freeze-Dried Powder, Salvia Miltiorrhiza Extract, Ginkgo Biloba Extract, Poria Cocos Extract, Antler Powder, Radix et Rhizoma Ginseng Extract | G20170072 |
|  |  | Shaolin brand Huangqi Ginseng Deer Antler Pills | Ginseng, Astragalus, Angelica sinensis, Malus antler, Yellow essence, Starch | G20190310 |
|  |  | Yijianyuan brand American ginseng, horse antler, and yellow slices | Radix Rehmanniae Praeparata Extract, Fructus Lycium barbarum extract, antler velvet powder (irradiated), Herba Epimedii Extract, Raspberry Extract, Radix et Rhizoma Ginseng Extract, Microcrystalline Cellulose, Film Coated Premix (Hydroxypropylmethylcellulose, Glyceryl Triacetate, Titanium Dioxide, Cochineal Aluminum Precipitate, Lemon Aluminum Precipitate, Ferric Yellow Oxide, Talcum Powder), Polyvidone K30, Magnesium Stearate | G20190374 |
|  |  | Golden Hemp Cordyceps and Deer Antler Slices | Cordyceps Powder, Antler Powder, Radix et Rhizoma Ginseng Extract; Excipients: Sorbitol, Film Coating Agent (Hydroxypropyl Methyl Cellulose, Glyceryl Triacetate, Titanium Dioxide, Lemon Yellow Aluminum Starch, Talcum Powder), Magnesium Stearate | G20200006 |
|  |  | Kunzhilin brand deer antler, Ganoderma lucidum and Epimedium wine | Goji Berry, Succotash, Ganoderma Lucidum, Cynomolgus, Malus Vulgaris | G20200157 |
|  |  | Miaogong brand deer antler, Eucommia ulmoides, and Sanqi capsules | Panax ginseng extract, Rhizoma Polygonati Odorati, Cortex Eucommia Ulmoides extract, Hippophae Rhamnoides extract, Antler Powder, Microcrystalline Cellulose, Magnesium Stearate | G20200163 |
|  |  | Purple Gold Port brand Bat Moth Pseudomold Plum Blossom Deer Antler Goji Berry Tablets | Batrachytrium cinereum powder, Plum antler powder (irradiated), Fructus Lycium barbarum extract | G20210074 |
|  |  | Yijia Neng brand ginseng, deer antler, and lingzhi slices | Maca Powder (irradiated), Horse antler (irradiated), Ginseng Extract, Ganoderma Lucidum Extract, Taurine | G20220164 |
|  |  | Lilan brand American ginseng deer antler capsules | American ginseng extract, antler powder (irradiated) | G20220172 |
|  |  | Aofulai brand ginseng deer antler capsules | Horse antler powder (irradiated), Ginseng extract | G20220183 |
|  |  | Zhonghe Hongye brand deer antler and American ginseng slices | Antler powder (irradiated), Radix et Rhizoma Ginseng Extract | G20220217 |
|  |  | Youli brand deer antler and American ginseng wine | Astragalus, Fructus Lycium barbarum, Panax ginseng, Malus antler | G20220249 |
|  |  | Bomiao brand ginseng, horse antler, maca tablets | Rhizoma Polygonati Odorati Extract, Ginseng Extract, Maca Powder (irradiated), Malus Vulgaris Powder (irradiated) | G20220318 |
|  |  | Lu Xilai brand wolfberry, American ginseng, deer antler wine | Gynostemma, Rhizoma Polygonati Odorati, Fructus Lycium Chinense, Longan Meat, Cynostemma Ginseng, Lohan Fruit, Velvet Horse Antler | G20220324 |
|  |  | Runxintang brand ginseng deer antler arginine capsules | Maca powder (irradiated), Equisetum vulgare powder (irradiated), Ginseng extract, L-Arginine, Zinc gluconate | G20230072 |
|  |  | Jianlu Sheng brand deer antler slices | Horse antler (irradiated) | G20230116 |
|  |  | Luguanzhuang brand deer antler, deer bone, and astragalus wine | Astragalus, Epimedium, Hawthorn, Fructus Lycium barbarum, red deer bone, red deer antler | G20230123 |
|  |  | Rongda Brand Deer Antler Lingzhi Oral Liquid | Ganoderma lucidum | G20230125 |
|  |  | Deer Xilai brand deer antler wolfberry wine | Epimedium, Longan Meat, Goji Berry, Schisandra Chinensis, Deer Blood, Deer Antler | G20230129 |
|  |  | Deer Xilai brand deer antler and American ginseng wine | Astragalus, Yellow Essence, Goji Berry, Panax Ginseng, Malus Vulgaris | G20230133 |
|  |  | Yupu brand American ginseng and deer antler wine | Malus antler, Panax ginseng, white wine, purified water | G20230151 |
|  |  | Ruiyuan brand deer antler and Cistanche deserticola capsules | Horse antler powder, Bat moth mycelium powder, Cistanchis sinensis extract, Magnesium stearate, Magnesium stearate | G20230177 |
|  |  | Qinqi brand wolfberry, American ginseng, and deer antler capsules | Herba Epimedium, Fructus Lycium barbarum, Radix Angelicae Sinensis, Radix et Rhizoma Ginseng, Antler Powder (irradiated) | G20230243 |
|  |  | Kunzhilin Brand Deer Antler Lingzhi Huangjing Oral Liquid | Yellow Essence, Goji Berry, Cynomolgus, Ganoderma Lucidum, Malus Vulgaris | G20230263 |
|  |  | Healthy Dream Brand Ginseng Deer Antler Maca Tablets | Maca Powder (irradiated), Equisetum Versicolor (irradiated), Salvia Miltiorrhiza Extract, Ginseng Extract, Ginkgo Biloba Leaf Extract, Sorbitol, Polyvinyl K30, Sodium Carboxymethyl Starch, Silicon Dioxide, Magnesium Stearate | G20230267 |
|  |  | Daqing Deer Garden Brand Deer Antler Epimedium Wine | Epimedium, yam, goji berries, yellow essence, red deer bone, horse antler, honey, white wine, purified water | G20230334 |
|  |  | Jiyun brand deer antler powder capsules | Malus antler powder | G20230557 |
|  |  | Federal Longfengbao brand ginseng, horse antler, maca capsules | Maca Powder (irradiated), Horse antler (irradiated), Ginseng Extract, Epimedium Extract, Chive Seed Extract | G20230654 |
|  |  | Bencao Zhengyuantang brand deer antler and American ginseng slices | Antler Powder (irradiated), Cucurbit extract, Microcrystalline cellulose, Maltodextrin, Sodium carboxymethyl starch, Magnesium stearate, Coating Powder (Hydroxypropylmethylcellulose, Glyceryl triacetate) | G20230717 |
|  |  | Yiyuan Brand Western Ginseng Deer Antler Monkey Mushroom Capsules | Monkey Head Mushroom, Gojia Pi, Goji Berry, Panax Ginseng, Malus Vulgaris | G20230761 |
|  |  | Jinglanchun brand ginseng deer antler arginine tablets | Maca powder (irradiated), antler powder (irradiated), ginseng extract, L-arginine, zinc gluconate, dextrin, magnesium stearate, coating powder (hydroxypropylmethylcellulose, polyethylene glycol, povidone K30) | G20230776 |
|  |  | Sanwang brand ginseng deer antler wine | Goji berries, ginseng, Epimedium, antler velvet, cloves, sand nuts, purified water, sugar, honey, white wine | G20240161 |
|  |  | Sea Saint brand American ginseng deer antler wine | Acanthopanax, Fructus Lycium barbarum, Herba Epimedium, Malus antler, Panax ginseng | G20240211 |
|  | Muli | Haiwang Brand Golden Oyster Capsules | Ostreae Concha powder, Amylum | G20040491 |
|  |  | Hongyangshen Brand Chitosan Oyster Tablets | Chitosan, Ostreae Concha extract | G20141320 |
|  |  | Golden Olive Brand Oyster Taurine Vitamin C Capsules | Ostreae Concha extract, Taurine, Vitamin C | G20150237 |
|  |  | Kemper Brand Sea Cucumber Oyster Capsules | Sea cucumber extract, Ostreae Concha extract | G20160459 |
|  |  | Chinese Salamander Brand Marine Fish Oligopeptide Oyster Oral Liquid | Marine fish oligopeptide powder, Ostreae Concha extract, Blueberry extract, Citric acid, Pectin, Sucralose, Ethyl Maltol, Purified water | G20200193 |
|  |  | Xuelong Brand Oyster Capsules with Sea Cucumber | Ostreae Concha extract, Sea cucumber powder, Lycii Frustus extract | G20230110 |
|  | Xuanshen | Ruinian Brand Xuan Ginseng and Western Ginseng Amino Acid Capsules | Silkworm pupa composite amino acid powder, Scrophulariae Radix extract, Panacis Quinquefolii Radix extract | G20141001 |
|  |  | Hop Fai Brand Xiyangshen Xuan Shen Sheng Di Huang Granules | Scrophulariae Radix extract, Rehmanniae Radix extract, Ophiopogonis Radix extract, Panacis Quinquefolii Radix extract, Dextrin, Stevia sugar | G20230352 |
|  |  | Xinzhu Nutritional Brand Di Huang Xuan Shen Tablets | Rehmanniae Radix, Scrophulariae Radix, Fritillariae Thunbergii Bulbus, Moutan Cortex, Menthae Haplocalycis Herba, Ophiopogonis Radix, Glycyrrhizae Radix Et Rhizoma, White granulated sugar, Povidone K 30, Carboxymethylstach Sodium, Magnesium stearate | G20230698 |
|  |  | Hop Fai Brand Xiyangshen Xuan Ginseng Sheng Di Huang Tablet | Scrophulariae Radix extract, Rehmanniae Radix extract, Ophiopogonis Radix extract, Panacis Quinquefolii Radix extract, Microcrystalline Cellulose, Magnesium stearate | G20230703 |
|  |  | Hop Fai Brand Xiyangshen Xuan Ginseng Sheng Di Huang Capsules | Scrophulariae Radix extract, Rehmanniae Radix extract, Ophiopogonis Radix extract, Panacis Quinquefolii Radix extract, Microcrystalline Cellulose | G20230704 |
| Maintaining healthy levels of serum lipid, and improving obesity | Juemingzi * | Baibang brand Lotus Leaf Cassia Seed Capsules | Cassia, Tea polyphenols, Ginkgo biloba, Lotus leaf, Starch | G20040410 |
|  |  | Hongyue Bencao Brand Danshen Fuling Cassia Seed Tea | Radix Angelicae Sinensis, Radix et Rhizoma Rhei, Radix Polygoni Multiflori, Poria Cocos, Salviae Miltiorrhizae, Semen Cassia Obtusifolia, Jujubae Fructus, Chrysanthemum, Black Tea | G20040733 |
|  |  | Xiangdan Qingpai Fuling Cassia Seed Tea | Radix Angelicae Sinensis, Radix et Rhizoma Rhei, Poria Cocos, Salviae Miltiorrhizae, Semen Cassia Obtusifolia, Radix Jujubae Nuttalli, Fritillariae Vulgaris, Chrysanthemum, Black Tea | G20040909 |
|  |  | Zhongyan Tongpai Juemingzi Gynostemma Tablets | Cassia, Gynostemma | G20050050 |
|  |  | Green Skinny Brand Cassia Seed Lotus Leaf Capsules | Semen Cassia, Job's tears, Semen Coix lacryma, Zelenium, Poria Cocos, Lotus Leaf, Pueraria Mirifica | G20050898 |
|  |  | Kangjiafu brand Cassia seed Ginkgo biloba leaf L-carnitine capsules | Cassia Seed, Lotus Leaf, Poria Cocos, Zeligia, Ginkgo Biloba, Wheat Germ Powder, L-Carnitine | G20060376 |
|  |  | Changqing Plan Brand Gynostemma pentaphyllum Cassia Seed Capsules | Gynostemma, Lotus Leaf, Cassia, Calcium Pyruvate, L-Carnitine | G20060403 |
|  |  | Yike Yuan brand Hongqu Hawthorn Cassia Seed Capsules | Red Currant, Hawthorn, Cassia Seed | G20060463 |
|  |  | Hongyue Bencao Brand Huomahen Konjac Cassia Seed Capsules | Lotus leaf, Poria cocos, Semen Cassia Obtusifolia, Semen Armeniacae, Polygonum Multiflorum, Oolong Tea Extract, Konjac Extract Powder | G20060653 |
|  |  | Liren brand L-carnitine cassia seed capsules | Poria cocos, Semen Cassia Obtusifolia, Senna Zedoaria, Lotus Leaf, Semen Coix lacryma, Citrus aurantium, L-Carnitine | G20070008 |
|  |  | Dianling brand Yuganzi Cassia Seed Capsules | Eugenol, Semen Cassia Obtusifolia, Semen Armeniacae, Lily, Gynostemma | G20080667 |
|  |  | Fengfengtang brand Danshen, Zexie, Goji, Cassia, and Hawthorn Capsules | Salvia miltiorrhiza, zelda, goji berries, cassia seeds, hawthorn, starch | G20090345 |
|  |  | Tiweishi brand L-carnitine cassia seed granules | Algae, Poria, Cassia, Gynostemma, Lotus Leaf, L-Carnitine | G20100449 |
|  |  | Yisheng Tongwo brand Pu'er Cassia Seed Lotus Leaf Tea | Pu'er Tea, Cassia, Lotus Leaf, Hawthorn, Poria Cocos | G20100484 |
|  |  | Desheng Huimin brand Gynostemma pentaphyllum cassia seed lotus leaf tea | Lotus leaf, green tea, cassia seed, zelda, gynostemma | G20110136 |
|  |  | Jinyuan brand Gynostemma pentaphyllum, Cassia seed, Lotus leaf tea | Lotus leaf, green tea, cassia seed, zelda, gynostemma | G20110136 |
|  |  | Qianquan Brand Cassia Seed Alisma Leaf Tea | cassia seed, zelda, oolong tea (irradiated), lotus leaf (irradiated), gynostemma | G20120004 |
|  |  | Weijian brand Cassia seed lotus leaf capsule | cassia seed, ginkgo biloba, zelda, gynostemma, lotus leaf | G20130046 |
|  |  | Huoshengyuan brand L-carnitine, lotus leaf, cassia seed, poria cocos, and diarrhea capsules | Lotus Leaf Extract, L-Carnitine Tartrate, Cassia Seed Extract, Poria Cocos Extract, Zelda Extract, Starch, Magnesium Stearate | G20130496 |
|  |  | Bidi brand hawthorn, cassia seed, lotus leaf tea | Green Tea (irradiated), Hawthorn Extract, Coix lacryma Extract, Semen Cassia Obtusifolia Extract, Green Tea Extract, Zea Mays Extract, Lotus Leaf Extract | G20140676 |
|  |  | Fuzhen brand L-carnitine tea polyphenol cassia seed lotus leaf capsule | Cassia Seed Extract, Lotus Leaf Extract, Tea Polyphenols, L-Carnitine, Magnesium Stearate | G20141178 |
|  |  | Hongyangshen brand lotus leaf, Alisma, Cassia seed extract tablets | cassia seed extract, lotus leaf extract, zelda extract, oolong tea extract | G20150208 |
|  |  | Saint Niff brand Cassia seed lotus leaf tea | Cassia, Lotus Leaf, Pu'er Tea, Zeleny, Gynostemma, Lohan Fruit | G20160083 |
|  |  | Tianzhiyuan brand Cassia seed Gynostemma pentaphyllum oral solution | Cassia, Pueraria Mirifica, Hawthorn, Gynostemma, Lotus Leaf, Zelda | G20210065 |
|  |  | Miaoyu brand Cassia seed Poria cocos beverage | Cassia seed extract (irradiated), Poria Cocos Extract (irradiated), Zea Mays Extract (irradiated), Lotus Leaf Extract (irradiated), Apple Cider Vinegar, Apple Juice Concentrate, Hawthorn Juice Concentrate, Mel (honey), Sucralose (sucralose), Flavors (food), Purified Water (water). | G20230886 |
|  |  | Yishui Dan brand L-carnitine cassia seed capsules | Cassia, Gynostemma, L-Carnitine Tartrate, Lotus Leaf, Microcrystalline Cellulose, Magnesium Stearate | G20240313 |
|  |  | Kang'enbei brand lotus leaf, Poria cocos, Cassia seed tea | Green Tea (irradiated), Cassia, Poria, Hawthorn, Lotus Leaf, L-Carnitine Tartrate | G20240317 |
|  |  | Runxintang brand carnitine cassia seed tablets | Cassia Officinalis Extract, L-Carnitine Tartrate, Zelenium Extract, Lotus Leaf Extract, Astragalus Membranaceus Extract, Dextrin, Magnesium Stearate, Coating Powder (Iron Oxide Brown, Talcum Powder, Hydroxypropyl Methylcellulose, Polyvidone K30, Polyethylene Glycol) | G20240320 |
|  |  | Sunflower brand oolong tea, Cassia seed, Gynostemma pentaphyllum tea bag | Oolong tea (irradiated), Semen Cassia Obtusifolia, Gynostemma, Zeleno, Lotus Leaf, Senna (irradiated) | G20240349 |
|  |  | Tangshan Beijian brand lotus leaf cassia seed tea bag | Green Tea, Cassia Officinalis Extract, Pericarpium Citri Reticulatae Extract, Zea Mays Extract, Lotus Leaf Extract, Gynostemma Gynostemma Extract | G20240350 |
|  |  | Green Skinny Brand Cassia Seed Lotus Leaf Alisma Beverage | Cassia Seed, Lotus Leaf, Zea Mays, Potato Extract, Citric Acid, Citrus Extract, Orange Flavor, Sucralose, Purified Water | G20240361 |
|  |  | Guozhen Brand Bamboo Leaf Flavonoids Cassia Seed Tablets | Bamboo Flavonoids, Cassia Obtusifolia Extract, Grapefruit Powder, Sodium Ascorbate, Microcrystalline Cellulose, Sugar, Film Coated Premix (Hydroxypropylmethylcellulose, Povidone K30, Polyethylene Glycol 6000, Lemon Yellow Aluminum Precipitate, Brilliant Blue Aluminum Precipitate), Sodium Carboxymethystarch, Magnesium Stearate | G20240371 |
|  |  | Yishui Dan brand hawthorn cassia seed red yeast granules | Hawthorn, Cassia Seed, Zea Mays, Allium Sativum, Lotus Leaf, Red Yeast Powder, Dextrin, Sucrose | G20240384 |
|  | Kunbu, Juemingzi | Haiku Brand Cassia L-Carnitine Capsules | Cassiae Semen, Poria, Laminariae Thallus, Chitosan, L-carnitine, Amorphophallus konjac powder, Spirulina powder, Margarita | G20070249 |
|  |  | Jinjiaoli Brand Lotus Leaf Cassia Seed and Polygonum Multiflorum Capsules | Polygoni Multiflori Radix extract, Nelumbinis Folium extract, Cassiae Semen extract, Alismatis Rhizoma extract, Laminariae Thallus extract | G20100283 |
|  |  | Huanghong Brand Perilla Seed Cassia Seed Angelica Dahurica Capsules | Perillae Fructus, Cassiae Semen, Angelicae Dahuricae Radix, Laminariae Thallus, Coicis Semen | G20100547 |
|  | Lurong | Zhangzisong Brand Antler and Ginseng Oral Liquid | Ginkgo Folium, Hawthorn, Alismatis Rhizoma, Ginseng Radix Et Rhizoma, Cervi Cornu Pantotrichum | G20140455 |
| Maintaining healthy levels of blood pressure | Juemingzi | Jinnuo Tong Brand Astragalus Danshen Cassia Seed Capsules | Astragali Radix, Salviae Miltiorrhizae Radix Et Rhizoma, Ginkgo Folium, Puerariae Lobatae Radix, Cassiae Semen, Chrysanthemi Flos, Hippophae Fructus | G20100311 |
|  |  | Tian Keng Brand Rhizoma Pinelliae Cassiae Cortex Eucommiae Capsules | Apocyni Veneti Folium, Eucommiae Cortex, Cassiae Semen, Hawthorn | G20120667 |
|  |  | Jinnuo Tong Brand Astragalus Danshen Cassia Seed Capsules | Astragali Radix, Salviae Miltiorrhizae Radix Et Rhizoma, Ginkgo Folium, Puerariae Lobatae Radix, Cassiae Semen, Chrysanthemi Flos, Hippophae Fructus | G20240224 |
| Maintaining healthy levels of glucose | Haizao | Fumitra Brand Sodium Alginate Chromium Yeast Powder | Sodium alginate, Calcium chloride, Chromium yeast, Sodium citrate, Silicon dioxide | G20240134 |
|  | Juemingzi | Hong Kong Sang Brand Ginseng Cassia Seed Yellow Essence Oral Liquid | Ginseng Radix Et Rhizoma, Schisandrae Chinensis Fructus, Cassiae Semen, Ligustri Lucidi Fructus, Salviae Miltiorrhizae Radix Et Rhizoma, Polygonati Rhizoma, Lycii Frustus, Puerariae Lobatae Radix, Jujubae Fructus | G20080612 |
|  | Lurong | Camphor Pine Brand Deer Antler Maitake Oral Liquid | Astragali Radix, Rehmanniae Radix Praeparata, Ophiopogonis Radix, Dioscoreae Rhizoma, Cervi Cornu Pantotrichum | G20140566 |
|  | Xuanshen | Tongrentang Brand Astragalus and Ginseng Tea | Mori Cortex, Fructus Mori, Astragali Radix, Mori Folium, Natrii Sulfas Exsiccatus, Chromium Picolinate | G20130058 |
| Auxiliary protection from chemical liver injury | Haizao | Blue Key Brand Sodium Alginate Taurine Capsules | Sodium alginate (irradiated), Taurine (irradiated) | G20070380 |
|  | Juemingzi | Herbalife Brand Cassia Seed Panax ginseng and Fructus Schisandrae chinensis Capsules | Cassiae Semen extract, Notoginseng Radix Et Rhizoma extract, Panacis Quinquefolii Radix extract, Schisandrae Chinensis Fructus extract | G20110448 |
|  |  | Yipintang Brand Cassia Seed, Green Peel and Pericarp Tea | Cassiae Semen, Citri Reticulatae Pericarpium, Citri Reticulatae Pericarpium Viride | G20240309 |
|  | Muli | Haiwang Jintu Brand Oyster Soy Peptide Carnitine Oral Liquid | Purified water, Ostreae Concha extract, Soybean peptide powder, L-carnitine, Taurine, Zinc gluconate, Vitamin B6, Vitamin C, Fructose syrup, Honey, Soluble dietary fiber, Beta-cyclodextrin, Peppermint flavoring | G20110347 |
|  |  | Shanggong Qiangshengtang Brand Oyster, Pueraria dulcis and Hovenia dulcis Capsules | Puerariae Lobatae Radix extract, Auranth Fructus Immaturus extract, Schisandrae Chinensis Fructus extract, Ostreae Concha powder, Tea polyphenols, Microcrystalline cellulose | G20190159 |
